# Speech Detection via Respiratory Inductance Plethysmography, Thoracic Impedance, Accelerometers, and Gyroscopes: A Machine Learning‐Informed Comparative Study

**DOI:** 10.1111/psyp.70021

**Published:** 2025-02-14

**Authors:** Melisa Saygin, Myrte Schoenmakers, Martin Gevonden, Eco de Geus

**Affiliations:** ^1^ Department of Biological Psychology VU Amsterdam Amsterdam the Netherlands; ^2^ Amsterdam Public Health Research Institute Amsterdam UMC Amsterdam the Netherlands

**Keywords:** accelerometer, ambulatory assessment, heart rate, heart rate variability, respiration, speech detection, stress, wearable

## Abstract

Speech production interferes with the measurement of changes in cardiac vagal activity during acute stress by attenuating the expected drop in heart rate variability. Speech also induces cardiac sympathetic changes similar to those induced by psychological stress. In the laboratory, confounding of physiological stress reactivity by speech may be controlled experimentally. In ambulatory assessments, however, detection of speech episodes would be necessary to separate the physiological effects of psychosocial stress from those of speech. Using machine learning (https://osf.io/bk9nf), we trained and tested speech classification models on data from 56 participants (ages 18–39) under controlled laboratory conditions. They were equipped with privacy‐secure wearables measuring thoracoabdominal respiratory inductance plethysmography (RIP from a single and a dual‐band set‐up), thoracic impedance pneumography, and an upper sternum positioned unit with triaxial accelerometers and gyroscopes. Following an 80/20 train‐test split, nested cross‐validations were run with the machine learning algorithms XGBoost, gradient boosting, random forest, and logistic regression on the training set to get generalized performance estimates. Speech classification by the best model per method was then validated in the test set. Speech versus no‐speech classification performance (AUC) for both nested cross‐validation and test set predictions was excellent for thorax–abdomen RIP (nested cross‐validation: 96.6%, test set prediction: 98.5%), thorax‐only RIP (97.5%, 99.1%), impedance (97.0%, 97.8%), and accelerometry (99.3%, 99.6%). The sternal accelerometer method outperformed others. These open‐access models leveraging biosignals have the potential to also work in daily life settings. This could enhance the trustworthiness of ambulatory psychophysiology, by enabling detection of speech and controlling for its confounding effects on physiology.

## Impact Statement

Speaking, through its unique respiratory demands, affects fundamental physiological parameters including heart rate and its variability. If not detected and controlled for in research in naturalistic settings, speech distorts the interrelationships between physiological reactivity and psychological constructs. Utilizing supervised machine learning, we validated and compared biosignal‐based speech detection models for respiratory inductance plethysmography, impedance, and accelerometer signals recorded with wearables. All models showed excellent performance and can help mitigate the confounding effects of speech activity.

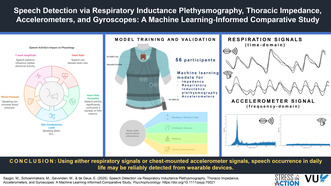

## 1 Introduction

The field of psychophysiology is well‐served by incorporating daily life measurements alongside traditional laboratory‐based research. Daily life measures are typically recorded through portable and lightweight wearables with attached or embedded sensors, or in the case of ecological momentary assessment (EMA), through mobile applications (Bertz et al. 2018; Kubiak and Stone 2012; Trull and Ebner‐Priemer 2013). This approach generates continuous physiological data coupled with repeated assessments of external events, psychological states, and behaviors over the day. Thus, it becomes possible to study the dynamics of physiological reactivity in relation to different stressors along with the cognitions and emotions they induce within a free‐living environment. This increases ecological validity and reduces recall bias (Trull and Ebner‐Priemer 2014), facilitating the identification of which psychological factors and physiological response patterns (peak reactivity, prolonged duration of reactivity, recovery, or habituation) contribute to allostatic load (Epel et al. 2018; Smyth et al. 2023).

The advantages of ambulatory assessment of physiology come, however, with a major challenge: confounders that are typically well‐controlled in the laboratory cannot be controlled to the same extent in unsupervised, naturalistic settings. Physical activity and changes in posture are the primary examples of such confounding factors. The effects of such gross body movement on autonomic regulation of the cardiovascular system are typically orders of magnitude larger than reactivity to psychosocial stressors or changes in psychological state, and therefore tend to overwhelm them (Grossman et al. 2004; Wilhelm et al. 2006). Consequently, posture and physical activity have been widely recognized as the factors that should at least be accounted for when analyzing physiological responses to stressors in daily life data (De Geus and Gevonden 2022; Houtveen and De Geus 2009; Verkuil et al. 2016). Methods to deal with physical activity and changes in posture capitalize on the concurrent recording of these confounders by accelerometers, geolocation tracking, or electromyography data, sometimes supplemented with electronic diaries (Reichert et al. 2020; Wilhelm et al. 2003). While the emphasis on taking into account gross body movement is warranted, other potential confounders of ambulatory physiological recording have received less attention than deserved. Active speech production, a frequently recurring everyday activity, is a primary example of such potentially important but “forgotten” confounders. A reason for their lack of prominence possibly lies in a lack of valid and easily applicable methods for co‐recording these confounders in ambulatory paradigms.

The respiratory system is known to modify cardiovascular responses in a coupled fashion (Bernardi et al. 2001; Eckberg 2003; Quintana and Heathers 2014). For example, indices of respiratory sinus arrhythmia (RSA), such as high‐frequency heart rate variability (HF‐HRV), often used by psychophysiology researchers to indicate cardiac vagal control, as well as overall heart rate variability indices like SDNN, are significantly affected by changes in respiration (Grossman and Taylor 2007; Quigley et al. 2024; Ritz and Dahme 2006; Soer et al. 2021). Speech production results in respiratory behavior that is consistently distinct (i.e., faster inhalation and slower exhalation) from that seen during regular metabolic respiration (McFarland 2001). Yet, ambulatory studies rarely attempt to control for physiological effects of speaking. Failing to account for the effects of respiratory changes on heart rate variability indices among other parameters of cardiovascular activity can lead to misinterpretation and concealment of the true relationships between physiological reactivity, stressors, and health outcomes (Beda et al. 2007; Lynch et al. 1980). The neglect of speech effects on physiology is not limited to ambulatory assessment. Many lab studies use stress paradigms which require the participant to speak, including the Stroop Color‐Word Task (Stroop 1935), public speaking tasks such as defending oneself from an accusation of shoplifting (Cacioppo et al. 1994; Saab et al. 1989), or the Trier Social Stress Test (Kirschbaum et al. 1993). If physiology during these active speaking tasks is compared to baseline physiology when participants are silent, an important portion of the physiological “stress reactivity” could be attributable to the effects of speech rather than effortful coping with task demands (Het et al. 2009).

### 1.1 Effects of Speech on Psychophysiological Measures

Studies using speech tasks demonstrated potential confounding effects of speaking on many of the autonomic measures used in psychophysiology. Speech can lead to significant increases in heart rate (HR), peripheral vasoconstriction, systolic (SBP) and diastolic blood pressure (DBP), and decreases in T‐wave amplitude and pulse transit time. The magnitude of speech‐related changes for these measures can be close to, or even higher than, changes in response to mental stress tasks requiring active effortful coping (Bernardi et al. 2000; Friedmann et al. 1982; Liehr 1992; Linden 1987; Linden and Estrin 1988; Lynch et al. 1980). Performing low‐effort speech tasks have also been shown to heighten mean tonic skin conductance level, amplitude of skin conductance responses, and changes in peripheral blood pulse volume compared to opening and closing of the jaw (Arnold et al. 2014; Weber and Smith 1990). Levels of salivary alpha‐amylase, known to increase in response to acute stress (Nater et al. 2006), went up relative to a silent baseline when participants described what happened in a neutral video (Het et al. 2009). These findings are all congruent with speech‐induced activation of the sympathetic nervous system.

Cardiac vagal—parasympathetic—effects on the heart rhythm decrease during (early) inspiration, and increase during expiration (Berntson et al. 1993; Eckberg 2003; Yasuma and Hayano 2004). As respiration during speaking is characterized by shorter inspiration and longer expiration times (McFarland 2001), speech would be expected to alter physiological parameters related to cardiac vagal control. Indeed, indices (e.g., high‐frequency heart rate variability HF‐HRV) used to capture RSA significantly increased while reading text aloud compared to reading silently (Dodo and Hashimoto 2019; Reilly and Moore 2003). Mental stressor tasks that required speaking yielded higher RSA amplitude and SDNN than a silently performed stressor, despite both types of stressors being perceived as similarly stressful (Brugnera et al. 2018). Likewise, Sloan et al. (1991) showed that adding a speech component to a silent mental arithmetic task reverted the decrease in RSA amplitude. For an aloud math stressor task, RSA amplitude was closer to that of a neutral talking task than to a silent math stressor task (Beda et al. 2007). RSA amplitude also increased when participants, maintaining the same respiratory rate, adopted the typical breathing pattern (short inhalation, long exhalation) observed during speaking (Strauss‐Blasche et al. 2000). The increase in RSA indices during speech may be due to a much longer period for lengthening of the heart periods during the prolonged exhalation (Strauss‐Blasche et al. 2000). Because speaking inflates RSA indices during conditions that are expected to reduce cardiac vagal activity (Brugnera et al. 2018; Reilly and Moore 2003), their potential to index this activity is seriously compromised (Quintana and Heathers 2014).

The studies reviewed argue for caution in interpreting changes in autonomic nervous system measures to reflect psychological stress when changes in respiratory patterns, such as those induced by speech, are not controlled for. While laboratory studies can deal with this problem through choices in the experimental design, this is not an option in ambulatory paradigms. Separating the physiological effects of speaking from those of psychological stress can only be done analytically in naturalistic studies, and would require the continuous registration of speech episodes.

### 1.2 Microphone‐based Methods for Speech Detection

The most accessible method to acquire continuous speech data in ambulatory assessment is to access the microphone of mobile phones (Soundsense by Feng et al. 2018; TILES by Lu et al. 2009). While this reduces participant burden by not requiring an extra device, it introduces considerable privacy concerns, as it records not only own speech but also that of individuals nearby. Employed adults indicated a dislike for audio recording, perceived it as intrusive, and experienced discomfort even when speech could not be reconstructed from the recordings (Klasnja et al. 2009). Intermittent recording methods can also be used, such as done by the EAR unit, capturing raw audio for short durations (e.g., 50 s) during a portion of the day (e.g., 5%) for natural conversation sampling with improved privacy sensitivity (Mehl 2017). Such intermittent sampling, however, does not allow for continuously recorded physiological changes to be corrected for concurrent speech production. It also poses considerable legal, ethical, and logistical challenges to researchers such as requiring all parties involved in a conversation to provide consent, as is the case in some US states (for a review, see Robbins 2017).

Apart from privacy concerns, microphones can easily get occluded by objects (e.g., placement in pocket for regular microphones, contact with clothing collar for throat microphones), and require laborious and error‐prone processing steps to differentiate background noise and speech from the participants' own speech (Rahman et al. 2011). To combat this issue, the Sociometric Badge (Olguin et al. 2006) used a microphone badge to record frequency, pitch, and amplitude of speech to capture face‐to‐face interaction durations (Ito‐Masui et al. 2021; Yu et al. 2016). To detect speech presence, the OlMEGA system used the synchrony of symmetrically placed binaural microphones attached to a pair of glasses (Pohlhausen et al. 2022). Due to their visibility, however, these wearables in badge or glasses form may have low aesthetic acceptability and reduce compliance (Rahman et al. 2011).

### 1.3 Biosignal‐based Methods for Speech Detection

A noninvasive method that circumvents many of the aforementioned concerns is detection of speech based on changes in respiratory waveform features. Various ambulatory respiration measurement methods use sensors that can be placed under clothing, and out of sight. Previous studies using this method typically made use of a noninvasive single thoracic or multiple (e.g., thoracoabdominal) respiratory belts, followed by feature extraction and inputting those features into an algorithm for supervised training of machine learning models (Ejupi and Menon 2018).

Respiratory inductance plethysmography (RIP) belts are accepted as having better accuracy than other methods of ambulatory respiration measurement (e.g., strain gauge belts or impedance pneumography), due to the absence of cardiac artifacts in the signal and the ability to better capture tidal volume in a variety of (e.g., prone) postures (Brouillette et al. 1987; Sackner and Krieger 1989; Wilhelm et al. 2003). In RIP, a high‐frequency low‐voltage current gets sent through sinusoidal coils *encircling* the body, and as the rib cage (RC) and abdomen (AB) expands, the self‐inductance (resistance against the current flow) of the coil changes as a function of the cross‐sectional area encircled, proportional to lung or diaphragm volumes, and raw waveforms of breath cycles are generated (Zhang et al. 2012). RIP typically takes into account both degrees of freedom of the chest wall, as RC and AB can also move independent of one another (DalľAva‐Santucci and Armanganidis 1991).

Another noninvasive method to record respiration is through the impedance cardiogram. Combined with the electrocardiogram (ECG), impedance cardiogram has often been used to characterize the within‐subject effects of sympathetic activity on the heart through measures such as the preejection period, cardiac output, ventricular ejection time, and stroke volume (Sheikh et al. 2024; Van Der Mee et al. 2023). However, thoracic impedance changes can also be used to assess respiratory waveforms, a technique referred to as impedance pneumography (Ernst et al. 1999). The electrical impedance (ImP) across the thorax increases when the volume of air inside the thorax increases during inhalation and decreases again during exhalation. The resulting respiration signal yields the inspiration and expiration intervals, as well as the respiratory depth (Houtveen et al. 2006). Having access to simultaneous ECG and impedance‐based respiration also allows the calculation of peak‐valley RSA, making respiration recording from the impedance cardiogram particularly attractive for psychophysiological studies. Like RIP, ambulatory ImP devices can be placed under clothing without signal interference, and allow for privacy‐sensitive recordings over prolonged periods in daily life.

A further detection method for speech is the use of accelerometers and gyroscopes. These sensors are commonly used in ambulatory studies, come at low costs, can be positioned under clothing (Ortiz et al. 2019), and are considered nonobtrusive to the wearer (Klasnja et al. 2009). If accelerometers or gyroscopes are positioned close to the larynx (e.g., on the sternum), and if they sample at a sufficiently high frequency, the high‐frequency vibrations of the vocal folds can be detected. As the frequency range within which larynx vibrations lie are considerably higher than that for physical movement, changes in signal caused by physical activity can be reliably eliminated via signal filtering. Accelerometers are also privacy‐sensitive as they do not record the raw speech content.

To date, a number of studies have aimed to implement speech detection using respiratory belts with piezoresistive sensors (Ejupi and Menon 2018), respiratory inductance plethysmography (Rahman et al. 2011; Wilhelm et al. 2003), impedance pneumography (Ramakrishnan et al. 2017), and accelerometer positioned at the central sternum (Matic et al. 2012) or neck (Carullo et al. 2013; Dubey et al. 2020; Mehta et al. 2012; Ortiz et al. 2019). However, these studies tended to have very small sample sizes or validated their models with mostly sedentary laboratory tasks that do not reflect the variation in daily activities during which people produce speech, hampering generalizability. Importantly, a direct comparison of the speech classification performance of the different methods has not been performed as of date. Therefore, the question of which biosignal method should be the preferred choice for ambulatory speech detection remains unanswered.

### 1.4 Aims of the Current Study

Directly measuring all biosignals *and* using a reference device (e.g., throat microphone) in daily life for multiple days would be highly burdensome to the participant. The current study, therefore, uses a controlled laboratory approach to first identify the best‐performing biosignal method for speech detection which, in a next step, could then be validated in daily life settings. In an experiment allowing for a rigorous manipulation of the ground truth of speaking versus not speaking, we test for the individual speech detection performances of four biosignal‐based methods considered suitable for prolonged ambulatory assessment. We use a comprehensive procedure resembling different types of speech typically produced in daily life (neutral speaking, talking about matters important to the self, talking during physical exercise, and under mental stress). The four methods consist of respiratory inductance plethysmography (RIP) using the Hexoskin Proshirt (Carré Technologies Inc. San Francisco, CA, USA) with both thorax and abdomen respiration signals (Method 1), only thorax respiration (Method 2); impedance pneumography (ImP) using the VU‐AMS 5fs (Method 3, Part a; Houtveen et al. 2006) as well as using the VU‐AMS Core (Method 3, Part b); and upper sternum‐mounted unit of triaxial accelerometers and gyroscopes (Method 4) using the VU‐AMS Core (VU Ambulatory Monitoring Solutions, Amsterdam, Netherlands). We make use of supervised machine learning (ML) with different learning algorithms (XGBoost, Gradient Boosting, Random Forest, and Logistic Regression) to discriminate between speech and no‐speech periods. When training these classification models, the ground truth is the experimental manipulation of when participants are instructed to speak (or not), verified by a video recording. Following data collection, participants are randomly divided into a training and test set that are used for all methods. Per method, using the training data, a nested cross‐validation procedure is carried out to obtain a robust (positive biases minimized) generalized estimate of each method's classification performance, indexed by the mean area under the receiver operating characteristic curve (AUC), accuracy, sensitivity, and specificity. Per method, we then train a best single classification model, and further confirm its speech detection capacity on the previously unseen test set.

For both the nested cross‐validation and test set validation steps, all four methods are expected to reach at least acceptable performance in differentiating (AUC > 0.70) speech from no‐speech segments (Hosmer and Lemeshow 2000). At both of these steps, we directly compare the speech classification performances of the four methods, having recorded all signals simultaneously in the participants. Between‐methods classification performance comparisons will be made using AUC as the primary index of interest. This statistic effectively takes into account both sensitivity (portion of speech periods detected as speech) and specificity (portion of no‐speech periods detected as no‐speech), providing a reliable summary of a model's discriminative performance (Janssens and Martens 2020; Kumar and Indrayan 2011). A priori, the models trained with features of both thoracic and abdominal bands of RIP (Method 1) are hypothesized to provide the highest AUC, due to the method's capability of capturing both degrees of freedom of respiration used for speech production: lungs and diaphragm (Konno and Mead 1967). In short:

Hypothesis 1 *In nested cross‐validation (i.e., providing generalized unbiased performance estimates), models of each method have at least acceptable classification performance, as suggested by mean AUCs > 70.0%*.

Hypothesis 2 *In nested cross‐validation, the classification performance of the models using the thoracoabdominal (2‐bands) RIP method is significantly higher than that of the models from the other methods, as suggested by the lack of overlap in the 95% confidence intervals of their AUCs*.

Hypothesis 3 *In the classification of the unseen test set, the best single model of each method has at least acceptable classification performance (AUC > 70.0%)*.

Hypothesis 4 *In the classification of the unseen test set, the best single model of the two‐bands RIP method significantly outperforms (p < 0.01) the models of the other methods*.

## 2 Methods

### 2.1 Ethics Statement

The study was approved by the local ethics committee, VCWE‐2023‐087. The paper was preregistered on the Open Science Framework (https://osf.io/bk9nf). All participants provided informed consent.

### 2.2 Participants

The inclusion criteria were fluency in English and being at least 18 and at most 65 years old. Exclusion criteria were having a current diagnosis of a severe neurologic or psychiatric disease, cardiac rhythm disorder, severe asthma, or other pulmonary disease, and a current use of any antiasthmatic drugs, drugs influencing respiratory behavior or cardiac autonomic nervous system activity. Participants were recruited through the university's study participant pool management interface and study flyers posted across the campus, and reimbursed with €15 (44.6%) or research participation credits.

### 2.3 Procedure

#### 2.3.1 Wearable Set‐Up

**FIGURE 1:**
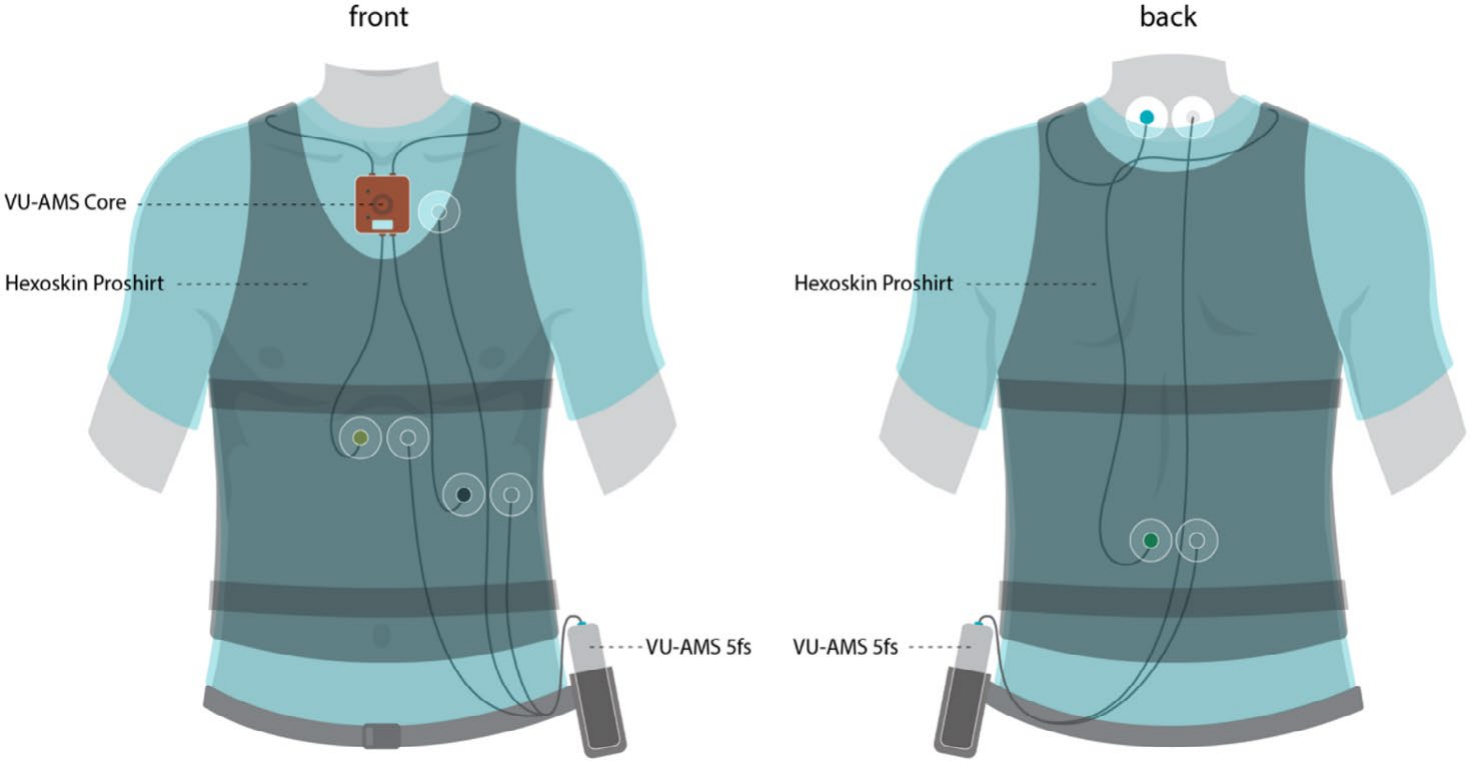
Wearable set‐up. Three electrodes (of each VU‐AMS 5fs and VU‐AMS Core) are located on the anterior side: (1) at upper sternum below the suprasternal notch, (2) at lower sternum on the xiphoid process, (3) at apex of the heart few centimeters below the xiphoid process. Two electrodes are placed on the posterior side: (4) a centimeter near the upper spinal cord, three centimeters above electrode 1, (5) a centimeter near the lower spinal cord, three centimeters below electrode 2. Hexoskin Proshirt with the thoracic and abdominal respiratory inductance plethysmography belts is worn on top.

Upon checking the eligibility criteria, the three wearables were set‐up (Figure 1), starting with VU‐AMS 5fs (https://vu‐ams.nl), an ambulatory monitoring device that simultaneously records electrocardiogram (ECG), impedance cardiogram (ICG), and triaxial accelerometer measurements (de Geus and Gevonden 2022; Nederend et al. 2017; Van Der Mee et al. 2023; Van Lien et al. 2011). It has five electrodes attached through wires to a central processing unit that is placed into a waist‐level belt. The wires were attached using H98SG adhesive hydrogel Kendall electrodes from Cardinal Health.

Next, we placed the VU‐AMS Core (5.5 × 5.5 × 1.5 cm), the successor device to VU‐AMS 5fs. In addition to ECG, ICG, and accelerometry (Acc), it can take triaxial gyroscope (Gyro) measurements. It was placed on the participant's upper sternum, a few centimeters below the suprasternal notch (Figure 1), using an adhesive Kendall electrode H98SG. The remaining electrodes were placed adjacent to those of VU‐AMS 5fs (Figure 1).

Finally, we fitted the Hexoskin Proshirt (Carré Technologies Inc., San Francisco, CA, United States), a smart garment previously validated against spirometry (Haddad et al. 2020; Montes et al. 2018; Villar et al. 2015) consisting of three dry ECG electrodes, respiratory inductance plethysmography (RIP) belts at the thorax and abdomen levels, and triaxial accelerometry at the waist level. The upper respiration belt was brought to the level of axilla, and the lower belt a few centimeters above umbilicus. Belts were tightened to capture the changes in bodily circumference.

#### 2.3.2 Experimental Protocol

To increase the likelihood that the speech detection methods from this laboratory experiment will be effective in ambulatory settings, we selected besides a silent baseline a varied set of speech tasks to emulate real world behaviors: reading a neutral text, semi‐structured conversation, reading while walking, and serial subtraction. Within each task (except for the baseline), we alternated between having participants speak aloud and having them generate an internal thinking process without vocalization to separate cognitive and physical task demands. To train models that can be used across posture changes, each task started while lying supine, continued in a sitting posture, and completed standing up, except for reading while walking (only performed standing) and serial subtraction (only performed sitting). Participants were later debriefed on the nature of the serial subtraction task, a mental stressor taken from the full Trier Social Stress Test (TSST) protocol (Kirschbaum et al. 1993). The entire procedure took around 1 h and 40 min.

##### 2.3.2.1 Silent Baseline

Participants were instructed to remain silent and move as little as possible. The recordings, repeated for supine, sitting, and standing posture, each lasted for 180 s.

##### 2.3.2.2 Reading Neutral Text

A text on the American River, retrieved from Wikipedia (“American River” 2023), was provided on paper. The text was selected to be neutral in content, describing the history, geography, and cultural practices of a region in California, USA. For each of the three postures, 90 s of reading aloud was followed by 90 s of reading silently as the participants held the paper in hand. As a manipulation check, to confirm participants actually performed the silent reading task, participants were asked at the end of each block what they remembered from the text they just read (aloud or silently).

##### 2.3.2.3 Semi‐Structured Conversation

Participants were then asked a total of 18 questions about themselves (e.g., “*What was the best gift you ever received and why*”) by the experimenter, 6 in each posture. Questions were taken from Aron and colleagues' inventory (Aron et al. 1997), specifically designed to build up interpersonal closeness through self‐disclosure to a stranger. Participants were instructed to give a continuous answer of at least 30 s in length (the end of which was indicated by a high‐pitch low‐volume auditory cue), and to keep answering continuously. Half of the questions were answered silently (in thought), and others aloud, in alternating order. During the aloud answers, the experimenter was actively listening and affirming via nonverbal cues. Once each 30‐s speech block was over the participants were encouraged to finish their answer. The silent answers represent episodes of internal speech where individuals think about personally relevant material.

##### 2.3.2.4 Reading While Walking

The participants walked on a treadmill set to a speed of 3.5 km/h, akin to fast walking. This speed was chosen so that the participants could simultaneously walk and read aloud without running out of breath. After an adjustment period of 120 s, they were handed back the neutral text, and completed another round of reading aloud and silently (continuing from where they left off), 90 s each. Following reading, they were asked what they remembered from the text. Subsequently, there was a recovery period of 2 min of seated rest before proceeding with the next task.

##### 2.3.2.5 Serial Subtraction Task

In seated position, participants had to repeatedly subtract the number seven starting from 907, alternating between subtracting aloud and subtracting silently every 30 s, for a total of 3 min. During the aloud segments, participants were instructed to start over when they made an arithmetic mistake. They were also told they were too slow and instructed to start over when they hesitated in their answering (retracting an answer or staying silent for longer than 3 s). The threshold for experimenter intervention was adjusted to participant skill level so that each participant was informed they were too slow and instructed to start over with a similar frequency. To monitor task compliance and to maintain stressfulness in the silent blocks, participants were asked at the end of each silent 30 s period to report the number they had reached. If they had completed fewer than 5 subtractions or the answer was not a valid number in the series, they were similarly told to start over.

### 2.4 Data Extraction, Synchronization, and Partitioning

The raw data for thoracic impedance (ImP) recordings, sampled at 250 Hz for VU‐AMS 5fs and 1000 Hz for Core, and the sternal accelerometer (Acc) and gyroscope (Gyro) signals (1000 Hz) were extracted via the VU‐DAMS software. The raw thorax and abdomen RIP data, sampled at 128 Hz each, were downloaded using HxConvertSourceFile, in EDF format. The data from all methods were read into a custom Python (version 3.11) script for synchronization and feature extraction. Firstly, the respiratory signals from Hexoskin (thorax RIP), VU‐AMS 5fs (first ImP), and VU‐AMS Core (second ImP) were used to synchronize the signals of the three devices via cross‐correlation. Signals were shifted in time until the cross‐correlations between the ImP signals and the thorax RIP signal were maximized. Any offset between the VU‐AMS Core ImP and the thorax RIP was applied to the Acc and Gyro data as well. The signal recordings were then all split into 30‐s segments, all laboratory conditions having lasted for a multiple of 30 s (e.g., 180 s of sitting baseline, 90 s of reading silently or aloud when walking). Sixty‐six 30‐s segments were thus retrieved per participant.

### 2.5 Signal Preprocessing for RIP and ImP

For the processing steps of RIP and ImP signals (both of VU‐AMS 5fs and VU‐AMS Core), the open‐source package *neurokit2* (Makowski et al. 2021) was utilized. Over every segment, a second order Butterworth bandpass filter was applied, with lower and upper thresholds of 0.05 to 0.60 Hz for thoracic and abdominal RIP, and with 0.05 to 0.55 Hz for impedance signals. Peaks and troughs of the respiratory waveforms were then detected in the segments with the peak‐detection algorithm of the *scipy* package (Virtanen et al. 2020) under *neurokit2*, setting the minimum time between peaks parameter (*peak_distance*) to 1.65 s. All peak‐trough detections were visually inspected. The upper thresholds for the specified signal bandpass filters were selected to maximize the accurate detection of peaks and troughs across the majority of participants. In occasional cases when the peak‐trough detections were not satisfactory (e.g., missed cycles due to high baseline respiration rate), the *peak_distance* parameter was adjusted for the given participant.

### 2.6 Feature Extraction for RIP and ImP

Respiration during speech is categorized by shorter inspiration time, longer expiration time, higher inspiratory flow, lower expiratory flow, along with increased variability in both respiration rate and inspiratory tidal volume (Abbasi et al. 2023; Beda et al. 2007; Fuchs and Rochet‐Capellan 2021). The inspiration duration along with its ratio to expiration time (IE ratio) have been identified as the primary respiratory markers that differentiate a speech period from silence (McFarland 2001; Wilhelm et al. 2003). Based on the literature, the following were all determined to be potentially important respiratory features to detect speech production: *mean IE ratio*, *standard deviation (SD) of IE ratio*, *mean expiratory time*, *SD of expiratory time*, *mean duty cycle* (inspiratory time over total cycle duration), *mean inspiratory time*, *SD of inspiratory time*, *inspiratory amplitude* (defined as the difference in *y*‐axis between a peak and its preceding trough), *inspiratory flow rate* (inspiratory amplitude over inspiratory time), *SD of inspiratory flow rate, respiratory rate variability or RRV* (calculated as root‐mean‐squared differences of breath to breath intervals, RMSSD), *SD of inspiratory amplitude*, *mean first difference of expiration* (differences between the expiratory times of consecutive breaths, averaged), *respiratory rate (RR)*, and *mean inspiratory minute amplitude* (total amplitude inhaled per minute, calculated as the mean inspiratory amplitude times the mean respiratory rate) (Beda et al. 2007; Bernardi et al. 2000; Binazzi et al. 2006; Fuchs and Rochet‐Capellan 2021; Hoit and Lohmeier 2000; Rahman et al. 2011; Wilhelm et al. 2003). *Expiratory amplitude*, *SD of expiratory amplitude, expiratory flow rate, SD of expiratory flow rate, and expiratory minute amplitude* were also extracted as the expiratory phase of speaking may illustrate noticeable decreases in volume flow (Conrad and Schönle 1979). An interesting feature, *peak‐trough symmetry* (originally proposed for neural oscillatory waves by Cole and Voytek 2019), was also added. As calibration against a spirometer was not performed for the respiration signals, we use the term inspiratory or expiratory *amplitude* instead of volume. Similarly, we calculate nonvolumetric flow rate values by dividing the *amplitude* over time (e.g., expiratory amplitude over time for expiratory flow rate).

For each of the 30‐s segments, the RR, RRV, and peak‐trough symmetry were extracted with the “interval‐related” feature function of *neurokit2*. The remaining features were extracted with our own code using the previously identified peaks and troughs. Occasionally, *neurokit2*'s interval‐related function gave a complexity embedding error or could not compute a peak‐trough symmetry feature, and the associated features would then be imputed *within*‐person, using the mean of other segments' values for the given feature. Thus, for each of the 66 observations of a participant, 21 features were derived for each of ImP VU‐AMS 5fs, ImP VU‐AMS Core, and single‐band thorax RIP, while 42 features were derived for that of the 2‐bands RIP.

### 2.7 Signal Preprocessing for the Accelerometer‐Gyroscope Unit

Fundamental frequency of voice, or the rate at which the larynx vibrates to facilitate speech production, ranges for adult biological men between 85 and 155 Hz, and for women between 165 and 255 Hz (Baken and Orlikoff 2000; Fitch and Holbrook 1970). To focus on the presence of these vibrations dispersed throughout the chest surface, over each 30‐s segment, a second order high‐pass Butterworth filter is applied at 75 Hz to all raw accelerometer (AccX, AccY, AccZ) and gyroscope (GyroX, GyroY, GyroZ) signals, using the *filtfilt* function from *scipy* (Virtanen et al. 2020). The *x*‐axis refers to the horizontal plane (medial‐lateral direction), the *y*‐axis to the coronal plane (superior–inferior direction), and the *z*‐axis to the sagittal plane (anterior–posterior direction).

### 2.8 Feature Extraction for the Accelerometer‐Gyroscope Unit

For each of the six sensors, spectral centroid, spectral bandwidth, spectral roll‐off (for 25%, 50%, 85% individually), spectral flatness, zero‐crossing rate, and root‐mean‐square were calculated over the 30‐s segments (30,000 samples per segment) using *librosa* (McFee et al. 2023), a Python package for audio analysis. *Spectral centroid* is the center of mass of the frequency spectrum (provided via Fast Fourier Transform of a time‐series data) of a signal. It is obtained by dividing the sum of weighted magnitudes (each frequency multiplied by its associated magnitude value) by the summed magnitude over all frequency bins. It has been commonly used in previous applications of voice activity detection (Mihalache and Burileanu 2022; Sreekumar et al. 2014). *Spectral bandwidth* (i.e., spectral spread, second moment of spectrum) is the standard deviation of distribution of frequencies around the spectral centroid, and has been used in previous audio classification studies (Mihalache and Burileanu 2022; Sandhya et al. 2020) as a fundamental descriptor of the frequency spectrum. Higher bandwidth indicates more divergence from the central tone, and lower spectral bandwidth indicates more convergence. Voiced segments are anticipated to have less spectral bandwidth. *Spectral roll‐off point* is the frequency below which a certain percentage (25%, 50%, 85% for the current paper) of all spectral energy of a given segment is contained, used in voice activity classification (Agarwal et al. 2022). *Spectral flatness* (tonality coefficient) is an index of peakiness of the frequency spectrum, calculated as the ratio of geometric mean over arithmetic mean of the power spectrum (Dubnov 2004), and can help detect speech presence (Ma and Nishihara 2013). Higher spectral flatness indicates a more uniform (less peaky) signal with regards to the power present at different frequency bins, better resembling white noise. *Root‐mean‐square energy* (*RMSE*) is a time‐domain feature representing the average intensity of the signal. Voiced speech introduces higher amplitude to the signal, making RMSE a typical feature for speech detection (Mihalache and Burileanu 2022; Pohlhausen et al. 2022; Verteletskaya and Sakhnov 2010). *Zero‐crossing rate*, a time‐domain feature, was among the first to be implemented in voice activity detection (Bachu et al. 2010; Graf et al. 2015; Pohlhausen et al. 2022; Rabiner and Sambur 1975), and represents the rate at which a time‐series signal changes from positive to negative and vice versa. Speech (i.e., a signal with higher periodicity) would have fewer zero‐crossings than white noise (Bachu et al. 2010; Sandhya et al. 2020). Using the *librosa* library, the abovementioned features were extracted in epochs of 2048 samples (2.048 s as the accelerometer and gyroscope sensors each has a sampling rate of 1000 Hz), and a new feature calculation over an epoch was initiated after every 512 samples (0.512 s). For each feature, the list of per‐epoch values provided by *librosa* were then simply averaged over the thirty‐second segment.

Additional features relevant for speech classification not provided by *librosa* were extracted by applying a Fast Fourier Transform (FFT) over each 30‐s segment for each of the 3 Acc and 3 Gyr signals. *Spectral entropy* has been widely used for voice activity detection (Graf et al. 2015; Misra et al. 2004) and was calculated via *scipy*'s entropy function with a base‐2 logarithm. The less sharp the peaks are in the FFT plot (akin to white noise), the higher the spectral entropy (Misra et al. 2004). *Spectral crest factor* is the ratio of the magnitude of the frequency bin (there is a frequency bin approximately every 0.033 Hz as sampling rate of the sensors is 1000 Hz and the number of samples entered into FFT is 30,000, providing sufficient frequency resolution) with the highest amplitude to the sum of all magnitudes in the spectrum, and was calculated using the function from the package *PyMIR*. *Spectral skewness* is the third order moment of the frequency spectrum and measures the symmetry near the centroid. *Spectral kurtosis*, a successful feature in speech detection (Zhang et al. 2009), is the fourth order moment of the frequency spectrum and gives an index of peakiness *around the centroid* (unlike spectral flatness which does not measure peakiness specifically around the centroid but considers the uniformity of power distribution across all frequencies). *Spectral variance*, expected to be lower for voiced segments, represents the variance of the magnitudes of the FFT bins, and unlike spectral bandwidth, is not weighted by the differences in frequency values (of each bin from the spectral centroid). *Spectral mean* is the sum of all magnitudes in FFT output divided by the number of frequency bins. Thus, for every segment, 84 features (14 from each of the six sensors) in total are extracted for the Acc‐Gyro (e) method. See the open‐access GitHub repository for the paper (see github.com/melisasaygin/SpeechDetection) and the Supplementary (Section 6) for the signal processing and feature extraction scripts as well as a written walkthrough.

### 2.9 Statistical Analyses

**FIGURE 2:**
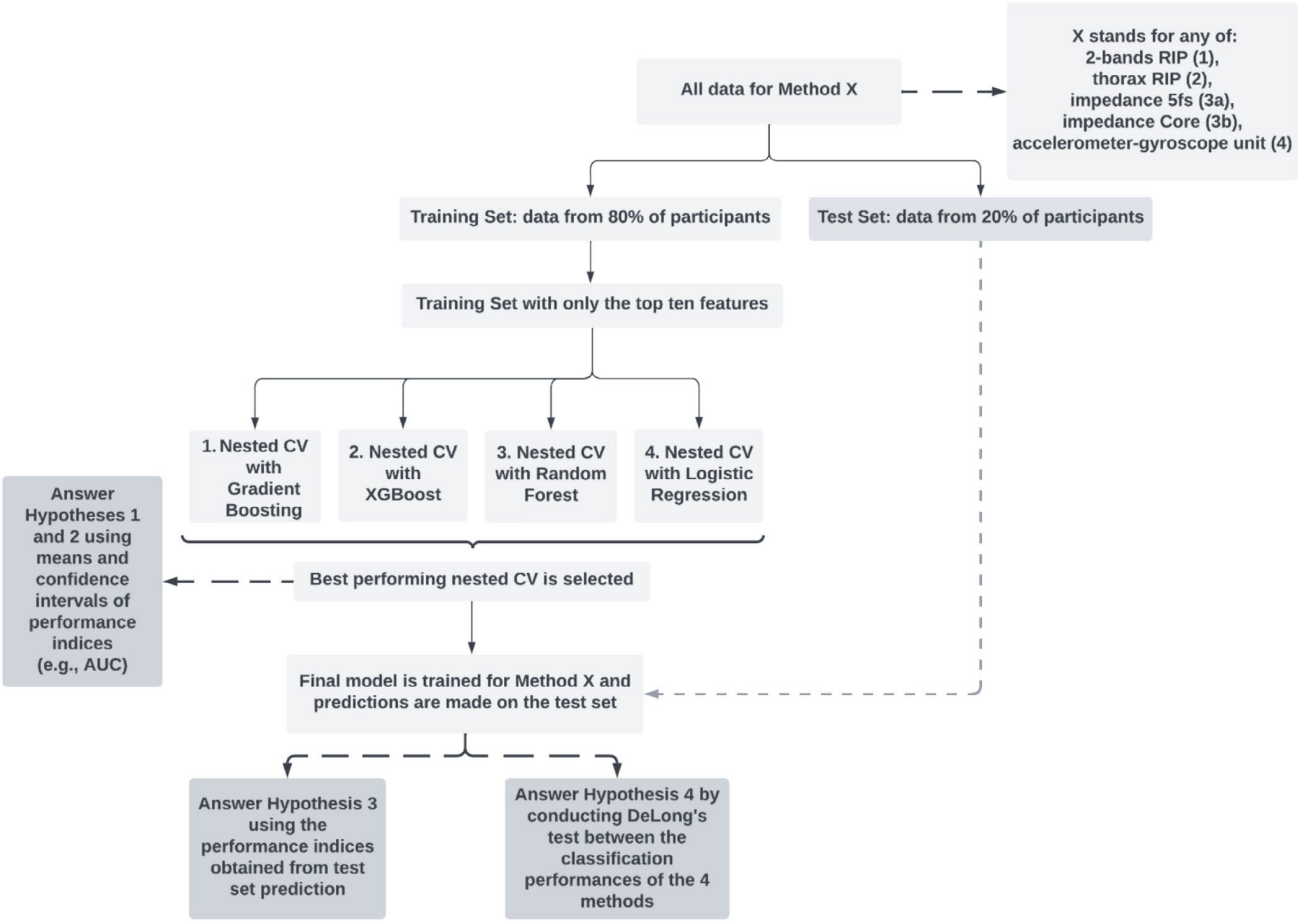
Machine learning analysis pipeline.

Twenty percent of participants were randomly picked as the test set (Figure 2). The features extracted from all four methods (1. 2‐bands RIP, 2. Thorax RIP, 3a. ImP VU‐AMS 5 fs, 3b. ImP VU‐AMS Core, and 4. Acc‐Gyro) coming from these individuals were not used for the nested cross‐validation part (Hypotheses 1 and 2). Per method, the extracted features of participants *except* the test set were placed into an .xlsx file along with their classifications (1 *= speech*; 0 = *no‐speech*), and read into a Python script (see the Supplementary, Sections 5 and 6 and the associated GitHub repository) with a machine learning (ML) pipeline utilizing *scikit‐learn* (Pedregosa et al. 2011).

Prior to starting the ML pipeline, the extracted features (e.g., 21 for thorax RIP, 42 for 2‐bands RIP, 21 for ImP, and 84 for Acc‐Gyro) of the *training* set along with the ground truth target (speech—no speech) were entered into the Mutual Information Gain (*mutual_info_classif*) function that calculates the dependency between a given feature and the target variable. If a feature is completely independent of speech presence, it would result in a mutual information gain of 0, and if opposite is the case, a gain of 1. To reduce computational burden and overfitting as well as to increase usability, the top 10 features (highest information gain) were picked for each method, and all others features for that method were discarded. We were thus left with 10 features per method that were used in the ML pipeline.

**FIGURE 3:**
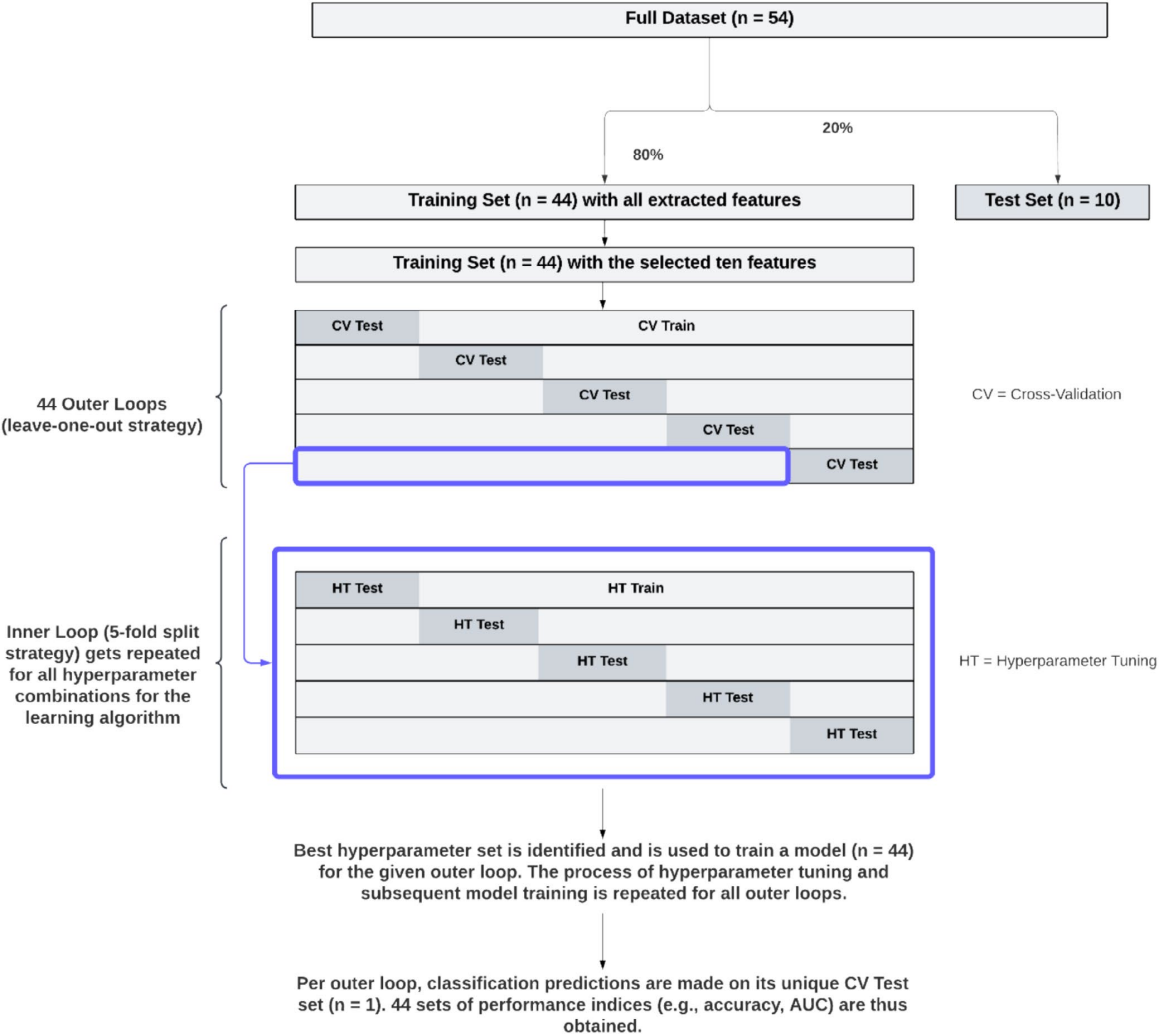
80/20 train‐test split followed by one nested cross‐validation, when starting with 54 participants' data.

For every combination of a method (e.g., 2‐bands RIP) and an ML algorithm (gradient boosting, XGBoost, random forest, and logistic regression), a nested cross‐validation (nested CV) was run (Figure 2). Nested CV can provide an unbiased estimate of how well a method's model would perform on average in reality (*the generalization estimate*), via the statistics of AUC, accuracy, sensitivity, and specificity. In a nested CV, multiple outer loops are created to train models using different subsets of the data, and test these on the remaining data. Specifically, the leave‐one‐out (LOO) approach was chosen as the outer loop strategy, in which each outer loop leaves out all of a participant's data (e.g., the 10 features belonging to the 66 segments for 2‐bands RIP method) as its CV test set (CV test in Figure 3), and trains a model using all remaining data (CV train) for that outer loop. This resulted in a number of outer loops equal to the number of participants entered into the nested cross‐validation. Performance statistics (e.g., AUC, accuracy) are ultimately averaged over all outer loop models.

In a nested CV, before training a model in each of these outer loops, hyperparameter tuning takes place for model optimization at the “inner” loops belonging to the outer loop. Specifically, a 5‐fold cross‐validation was implemented for all possible hyperparameter combinations. The optimal combination of hyperparameters that minimizes the mean test error is automatically picked, and only after, it is applied to the outer loop for training and later testing (on CV test). Though nested CV is a computationally expensive method, it decreases optimistic biases and overfitting that would have resulted from using the same data to both do hyperparameter tuning and evaluation of model performance (Cawley and Talbot 2010). See Figure 3 for a visual walkthrough of one nested cross‐validation (assuming a sample of 54).

For each method, the above illustrated process of a nested CV is repeated (Figure 2) using the following ML algorithms that are all suited for supervised binary classification: (1) gradient boosting (GB), (2) extreme gradient boosting (XGBoost), (3) random forest (RF), and (4) logistic regression (LR). Per method, the nested CV of the learning algorithm (e.g., nested CV in which XGBoost was used) that resulted in the highest averaged AUC (shortly, the best nested CV) was selected, and other nested CVs discarded. Using the results of this best nested CV of each method (Method 1, 2, 3a, 3b, and 4), both Hypothesis 1, questioning whether all methods can sufficiently (AUC > 70.0%) perform speech detection, and Hypothesis 2, questioning whether the models of the 2‐bands RIP method outperform those of other methods in general, can be answered. Hypothesis 1 is addressed via the AUC value (and as additional information, other performance indices such as accuracy, sensitivity, and specificity) averaged over the outer loops of a method's best nested CV. To address Hypothesis 2, the 95% Confidence Intervals (CI) of AUCs (*n* = 44) of the best nested CV are calculated per method. For any two methods to be deemed as significantly differing in their generalized classification performances, their 95% CIs need to be nonoverlapping.

As explained above, to get the generalization estimates per method, multiple models were created (one per outer loop) within a nested CV and their indices averaged. However, we also want *a single* best classification model per method, and test it to verify sufficient performance to support future dissemination to other researchers. Furthermore, having a best model per method will allow us to conduct statistical testing to compare between the different methods' classification performances. Thus, with all training data (≈80%), we train one model for each method, using the learning algorithm that was used in a given method's best nested CV (e.g., if XGBoost for Method 1 was the selected nested CV, XGBoost is again used while training this method's best model). The hyperparameters of a model also need to be specified prior to training. For each method, the hyperparameters of this single model were set to the mode (most frequently occurring values) across the outer loops of the best‐performing nested CV run.

Each method's best single model was then used to make predictions on the previously unseen test set (20%). The retrieved performance indices enable answering Hypothesis 3 by determining if the performance of each method's best model is sufficient (AUC > 70.0%) in classifying unseen test data. The prediction probabilities given by methods along with the true classification labels were then exported from Python and imported into an R script (see https://github.com/melisasaygin/SpeechDetection, the folder *Between‐Methods Comparison Using Test Set*) using the package *pROC* (Robin et al. 2011). To test Hypothesis 4 (that the 2‐bands RIP model outperforms the best models of other methods when tested on an unseen test set), we used DeLong's test (DeLong et al. 1988) to conduct statistical comparisons of the AUCs between the best model of each method (e.g., 2‐bands RIP versus thorax RIP). Significance level was set at *p* < 0.01.

## 3 Results

### 3.1 Sample Descriptives

The sample (*n* = 56, 75% female biological sex) was predominantly White European and had a mean age of 21.5 (SD = 4.42; range 18 to 39 years). Body mass index (BMI) was calculated separately for adults (20 years or older) and teenagers (19 and younger) using the directions of the Centers for Disease Control and Prevention. We found 81.8% of the sample to have healthy weight, while 9.09% classified as overweight, 5.45% as underweight, and 3.64% as having obesity. Average BMI was 21.8 kg/m^2^ (SD = 3.20, Range = [17.9, 31.8]). All participants at least attained a secondary school degree with some college coursework taken. Females (*M* = 267, 95% CI = [262, 272]) had significantly higher center frequency (calculated as spectral centroid at the anterior–posterior axis, in Hz) of speech than males (*M* = 232, 95% CI = [221, 243]).

### 3.2 Data Availability

For the Methods 1 and 2 (RIP), all data for both thorax and abdomen respiration was recorded without loss, meaning 66 30‐s segments of data per participant. For Method 3a (ImP VU‐AMS 5 fs), four participants' data files were not recorded due to memory card malfunctioning, and another participant had repeated electrode detachment, rendering the data invalid. With VU‐AMS Core, five participants' data were not recorded due to central processing unit errors, one participant's data was taken out due to low signal quality, and from another the first six 30‐s segments of data were removed due to a random noise pattern. There were two other participants whose data for all respiration‐based methods were removed (Method 1–3) due to unusual respiratory behavior at rest. The first indicated being trained in meditation and breathing exercises and had a respiration rate at rest that is significantly lower (e.g., 4 bpm) than typical, making the peak‐trough algorithm to detect breaths unreliably. The second showed atypical respiratory trends (i.e., unusually long pauses between breaths) evident in all respiratory recordings. Thus, there were 3564 30‐s segments (*n* = 54) each for thorax and abdomen RIP, 3234 (*n* = 49) for Impedance VU‐AMS 5 fs, 3162 (*n* = 48) for the Impedance of VU‐AMS Core, and 3366 (*n* = 51) for the Acc‐Gyro method deemed eligible for analysis. Within each participant's dataset, by experimental design, 36.4% of the 30‐s segments are speech periods (coded 1) and 63.6% no‐speech periods (coded 0). Thus, sufficient proportions of data representing both classifications were available to train the models.

### 3.3 Signal Quality

**FIGURE 4:**
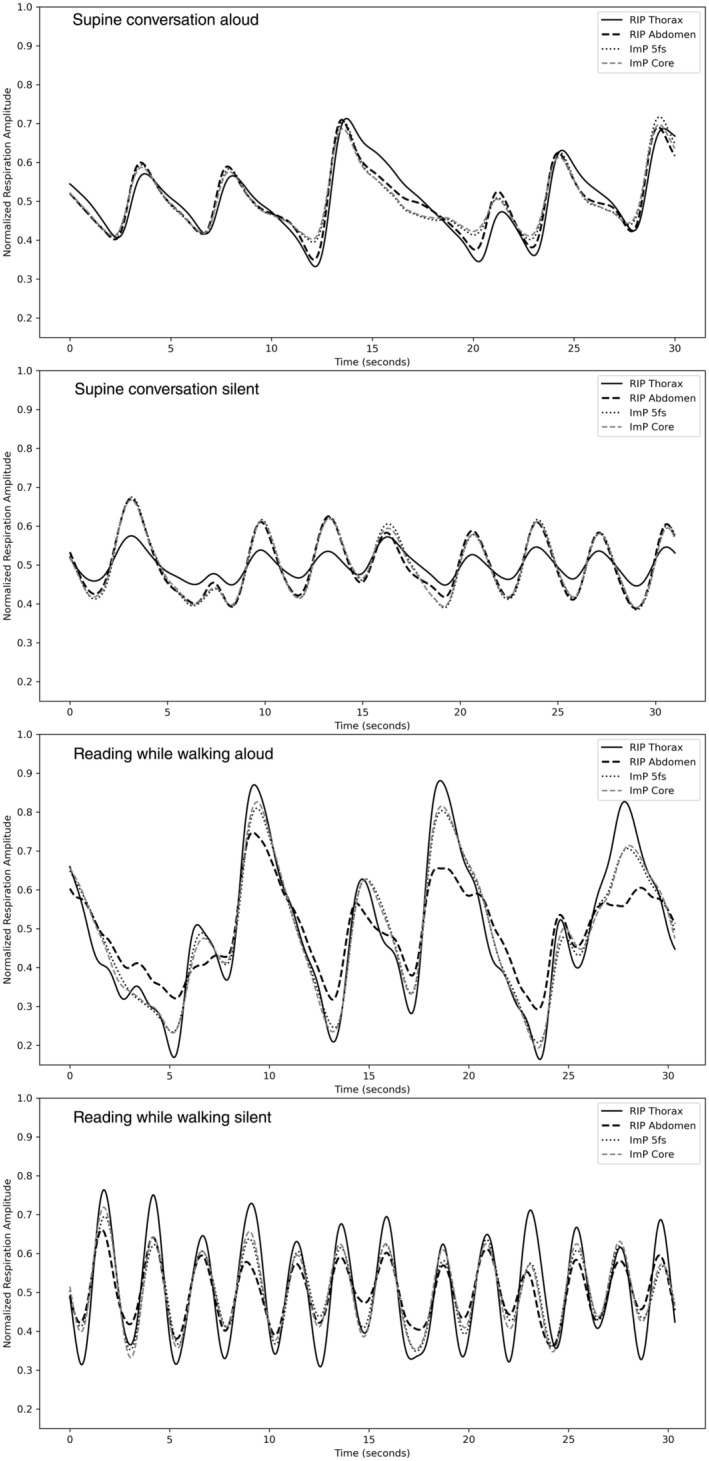
Visualization of the normalized respiratory signals for a participant over 30‐s segments from the supine conversation condition and the reading while walking condition from a representative participant.

Figure 4 displays the four simultaneously recorded respiration signals (RIP Thorax, RIP Abdomen, Impedance 5 fs, Impedance Core) of one participant for the conditions of supine conversation aloud, supine conversation silent, walking and reading aloud, and walking and reading silent. Each window represents a 30‐s segment. For visualization purposes, following cleaning, each signal was normalized over its entire recording duration. The values (of the time‐series) that deviated by more than 4.5 standard deviations from the mean were not taken into account during signal normalization. To normalize via min–max scaling, the minimum of an array was subtracted from each point, and the outcome was divided by the range (max ‐ min) of the array.

**FIGURE 5:**
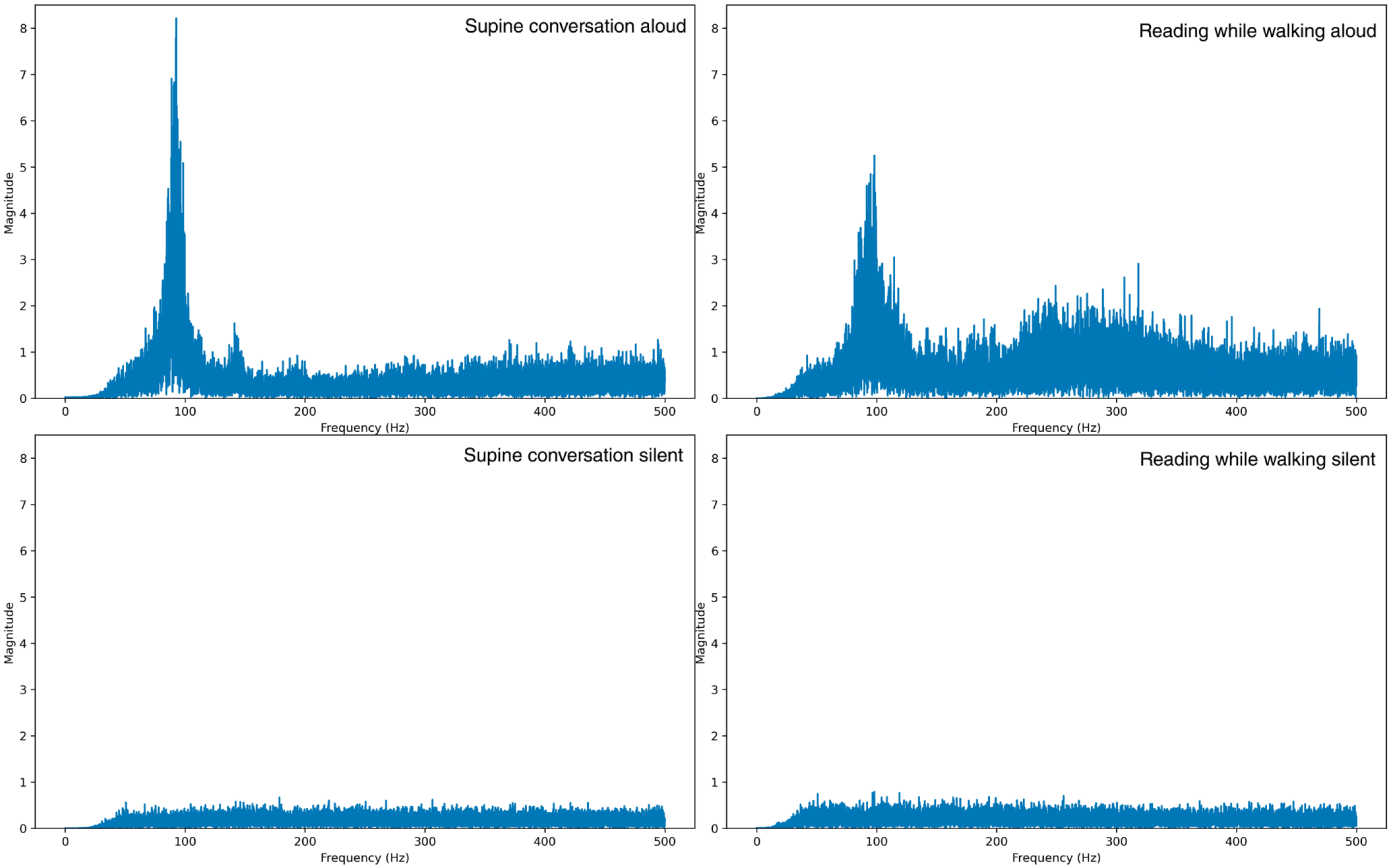
Magnitude versus Frequency content graphs of the accelerometer in sagittal plane from a representative participant over four segments, obtained following a Fast Fourier Transform.

Figure 5 displays the accelerometer signal in the sagittal (*z*‐axis) plane in frequency domain, for the same individual and conditions as above. After each 30‐s segment in time domain was high‐pass filtered, it was Fast Fourier Transformed. The magnitudes of each frequency bin within the segment are plotted up to a maximum of 500 Hz (half the sampling frequency of the accelerometer).

### 3.4 Physiological Descriptives

Table 1 shows the means and standard deviations of the respiratory rate (RR) and inspiratory to expiratory duration ratio (IE ratio) for the four different respiration signals recorded. This illustrates the effects of the posture, speech, and condition manipulations on respiratory behavior. The table also adds the heart rate (HR) recorded with VU‐AMS 5fs as an indication of the cardiovascular effects generated by these manipulations.

**TABLE 1:** Respiratory rate (RR) and inspiration to expiration duration (IE) ratio per task for all respiration signals.

	RIP thorax (*n* = 54)		RIP abdomen (*n* = 54)		Impedance core (*n* = 48)		Impedance 5 fs (*n* = 49)		
	RR	IE ratio	RR	IE ratio	RR	IE ratio	RR	IE ratio	HR
Supine baseline	15.6 (2.72)	0.860 (0.091)	15.6 (2.66)	0.853 (0.094)	15.7 (2.95)	0.904 (0.157)	15.6 (2.69)	0.862 (0.106)	67.2 (10.6)
Sitting baseline	16.0 (2.71)	0.823 (0.130)	15.8 (2.88)	0.795 (0.127)	15.8 (2.91)	0.893 (0.155)	16.0 (2.81)	0.869 (0.114)	76.8 (12.2)
Standing baseline	16.6 (3.35)	0.833 (0.118)	16.5 (3.42)	0.818 (0.128)	16.5 (3.24)	0.909 (0.157)	16.6 (3.30)	0.869 (0.133)	91.0 (14.2)
Supine reading aloud	14.6 (1.87)	0.613 (0.127)	14.9 (1.86)	0.598 (0.123)	15.0 (2.30)	0.796 (0.259)	15.0 (2.20)	0.709 (0.197)	73.9 (10.1)
Supine reading silent	19.2 (3.10)	0.883 (0.097)	19.2 (3.15)	0.878 (0.091)	18.7 (3.05)	0.967 (0.164)	18.8 (2.94)	0.895 (0.120)	67.1 (10.9)
Sitting reading aloud	14.5 (2.23)	0.649 (0.269)	14.3 (2.22)	0.642 (0.241)	14.1 (2.16)	0.788 (0.222)	14.4 (2.08)	0.757 (0.176)	79.4 (10.7)
Sitting reading silent	17.2 (3.25)	0.873 (0.158)	17.2 (3.14)	0.822 (0.121)	17.0 (3.41)	0.935 (0.237)	17.2 (3.38)	0.861 (0.131)	75.2 (10.6)
Standing reading aloud	14.6 (2.06)	0.682 (0.330)	14.6 (1.87)	0.734 (0.188)	13.9 (1.95)	0.820 (0.241)	13.9 (2.00)	0.774 (0.242)	89.1 (12.6)
Standing reading silent	18.1 (2.99)	0.891 (0.168)	17.9 (3.09)	0.856 (0.162)	17.8 (3.08)	0.923 (0.165)	18.0 (3.06)	0.878 (0.104)	86.8 (13.6)
Supine conversation aloud	14.2 (2.25)	0.595 (0.132)	14.6 (2.31)	0.586 (0.138)	14.9 (2.16)	0.789 (0.230)	15.1 (2.33)	0.721 (0.225)	75.4 (11.4)
Supine conversation silent	17.8 (3.23)	0.847 (0.106)	17.8 (3.26)	0.832 (0.108)	17.5 (2.92)	0.962 (0.215)	17.4 (3.02)	0.878 (0.163)	64.4 (11.1)
Sitting conversation aloud	14.0 (2.41)	0.684 (0.316)	13.7 (2.33)	0.612 (0.180)	14.7 (2.66)	0.882 (0.258)	14.3 (2.64)	0.821 (0.269)	81.7 (10.2)
Sitting conversation silent	16.1 (3.10)	0.831 (0.162)	15.9 (3.18)	0.809 (0.172)	15.9 (3.05)	0.909 (0.173)	15.9 (3.02)	0.913 (0.389)	71.1 (10.6)
Standing conversation aloud	14.1 (2.33)	0.669 (0.333)	14.4 (2.11)	0.745 (0.208)	14.4 (2.07)	0.843 (0.201)	14.3 (2.32)	0.799 (0.242)	89.0 (12.5)
Standing conversation silent	16.9 (3.19)	0.858 (0.159)	16.7 (3.22)	0.861 (0.211)	16.7 (3.01)	0.920 (0.188)	16.6 (3.06)	0.876 (0.174)	80.8 (13.7)
Walking reading aloud	15.8 (2.12)	0.716 (0.269)	16.5 (2.16)	0.793 (0.202)	15.5 (2.05)	0.811 (0.226)	15.1 (1.94)	0.793 (0.204)	97.2 (11.8)
Walking reading silent	25.0 (2.89)	0.992 (0.105)	24.6 (2.65)	0.994 (0.103)	23.6 (3.46)	1.02 (0.165)	25.1 (3.08)	0.972 (0.056)	95.9 (11.7)
Serial subtraction aloud	15.8 (2.28)	0.706 (0.247)	15.7 (2.03)	0.691 (0.193)	15.4 (1.89)	0.833 (0.212)	15.7 (2.11)	0.834 (0.209)	84.3 (11.9)
Serial subtraction silent	18.4 (3.38)	0.816 (0.157)	18.4 (3.45)	0.792 (0.127)	18.1 (3.45)	0.864 (0.130)	18.2 (3.56)	0.854 (0.150)	76.2 (12.0)

*Note:* In each cell, the mean value is denoted above the standard deviation. HR were calculated using the VU‐DAMS software. RR is reported in breaths per minute, and HR in beats per minute.

Abbreviation: HR, heart rate.

Speech significantly decreased both the respiration rate and the inspiration to expiration duration ratio, foreshadowing that respiration signals can indeed be sources of speech classification. For a complete report of the main and interaction effects from the performed repeated‐measures ANOVAs, see the Supplementary (Section 1, Supplementary Tables S1).

#### 3.4.1 Physiological Manipulation Checks: Effects of Speech, Posture, and Condition on Heart Rate

A Greenhouse‐Geiser adjusted three‐way 2 (speech presence) × 3 (posture) × 2 (condition) repeated‐measures ANOVA with reading and conversation as the conditions showed that speech activity (*M*_in thought_ = 74.2, *M*_out loud_ = 81.4), *F*_1, 48_ = 226, *p* < 0.001, $\eta_{p}^{2}$ = 0.825, posture (*M*_supine_ = 70.2, *M*_sitting_ = 76.8, *M*_standing_ = 86.4), *F*_1.2, 59.2_ = 115, *p* < 0.001, $\eta_{p}^{2}$ = 0.705, and condition (*M*_reading_ = 78.6, *M*_conversation_ = 77.1), *F*_1, 48_ = 15.7, *p* < 0.001, $\eta_{p}^{2}$ = 0.247, all had significant main effects on heart rate. Of note is the large effect size for speech activity. These results confirm that speaking has substantial cardiovascular effects even when the psychosocial stress component of speaking is minimized.

A 2 (speech presence) × 2 (condition) rm‐ANOVA with reading (while standing) and walking as the conditions showed that both speech presence (*M*_in thought_ = 91.3, *M*_out loud_ = 93.2), *F*_1.00, 48.0_ = 15.9, *p* < 0.001, $\eta_{p}^{2}$ = 0.249, and exercise (*M*_standing_ = 87.9, *M*_walking_ = 96.5), *F*_1.00, 48.0_ = 75.7, *p* < 0.001, $\eta_{p}^{2}$ = 0.612 had a significant main effect on heart rate.

A 2 × 2 rm‐ANOVA with reading (while sitting) and serial subtraction as the conditions showed that both speech production (*M*_in thought_ = 75.7, *M*_out loud_ = 81.8), *F*_1.00, 48.0_ = 200, *p* < 0.001, $\eta_{p}^{2}$ = 0.806, and condition (*M*_sitting_ = 77.3, *M*_serialsubtraction_ = 80.3), *F*_1.00, 48.0_ = 14.8, *p* < 0.001, $\eta_{p}^{2}$ = 0.236, had significant main effects on heart rate. Thus, the implementation of serial subtraction from the Trier Social Stress protocol worked as intended. Of note, the effect of speech on HR was stronger than that of the stress task. For a full report of the main and interaction effects on HR, see the Supplementary (Section 1, Supplementary Tables S7–S9).

### 3.5 Train‐Test Splitting

Among the participants who have *full* data across all methods (*n* = 44), 10 were randomly selected to be set aside as the test set. Thus, 10 were set aside and the training dataset consisted of 44 individuals' data for Thorax RIP, 44 for 2‐bands RIP, 39 for ImP 5 fs, 38 for ImP Core, and 41 for Acc‐Gyro. Only these training sets were used for testing Hypotheses 1 and 2.

### 3.6 Feature Selection for Use in Machine Learning Pipeline

For each method, mutual information gain per feature was calculated using the training set. The 10 features with the highest information gain statistics (i.e., those that reduced the entropy in the target variable the most) were selected. The features that did not make the cut were eliminated and are not used in the rest of the analyses. Note that for the Acc‐Gyro Method, no gyroscope features were present among the top 10, but the top 10 represented only the features taken from the *z*‐axis (sagittal plane, in the anterior–posterior direction) of the accelerometer perpendicular to the sternum. Thus, this method hereafter will be referred to as Acc only. The center frequency, or the spectral centroid feature, which was shown to be significantly different between males and females, did not rank among the top 10 features. See the Supplementary (Section 2, Supplementary Tables 10–14) for a list of the 10 selected features per method.

The within‐person mean imputation of features, necessitated by rare errors of the *neurokit2* package, affected fewer than 1% of the segments. For example, mean imputation was done for 0.898% of the segments for the thoracic respiratory rate variability feature (among the 10 selected features for both the Thorax RIP and 2‐bands RIP methods), 0.758% of the segments for the abdominal respiratory rate variability feature, and 0.433% for the impedance (VU‐AMS 5 fs) respiratory rate variability feature.

### 3.7 Hypothesis 1: Can All Four Methods Detect Speech?

A nested cross‐validation was conducted for each method and learning algorithm combination (see Table 2) to identify the best‐performing algorithm for each method. For the 2‐bands RIP (AUC = 96.6%) and the thorax RIP (AUC = 97.5%) methods, XGBoost resulted in the highest averaged Area Under the Curve. For the impedance VU‐AMS 5fs (AUC = 97.0%), impedance VU‐AMS Core (AUC = 92.1%) and the Acc (AUC = 99.3%), Gradient Boosting provided the highest AUC but the differences with XGBoost were small. Hereafter, the specified best‐performing algorithm for a given method will be used in evaluating its speech detection ability.

**TABLE 2:** Nested cross‐validation performance statistics per method for the different machine learning algorithms.

	Gradient boosting	XGBoost	Random forest	Logistic regression
	*M ± SD*	*M ± SD*	*M ± SD*	*M ± SD*
	95% CI	95% CI	95% CI	95% CI
Thorax RIP (*n* = 44)				
AUC	97.3% ± 4.03% [96.1%, 98.5%]	**97.5% ± 3.85%** **[96.3%, 98.6%]**	95.9% ± 4.45% [94.6%, 97.3%]	95.3% ± 6.93% [93.3%, 97.4%]
Accuracy	92.1% ± 6.44% [90.2%, 94.0%]	**92.6% ± 6.55%** **[90.7%, 94.5%]**	88.9% ± 7.97% [86.5%, 91.2%]	90.9% ± 7.35% [88.7%, 93.0%]
Sensitivity	90.9% ± 10.3% [87.9%, 94.0%]	**91.3% ± 10.9%** **[88.1%, 94.5%]**	93.6% ± 8.87% [90.9%, 96.2%]	87.0% ± 12.1% [83.4%, 90.6%]
Specificity	92.7% ± 7.15% [90.6%, 94.9%]	**93.3% ± 6.59%** **[91.4%, 95.3%]**	86.2% ± 11.8% [82.7%, 89.7%]	93.1% ± 8.67% [90.5%, 95.6%]
2‐bands RIP (*n* = 44)				
AUC	96.5% ± 4.00% [95.4%, 97.7%]	**96.6% ± 4.12%** **[95.4%, 97.8%]**	95.3% ± 5.10% [93.8%, 96.8%]	94.6% ± 7.35% [92.4%, 96.8%]
Accuracy	90.7% ± 7.20% [88.5%, 92.8%]	**90.6% ± 7.06%** **[88.5%, 92.7%]**	87.5% ± 8.39% [85.1%, 90.0%]	88.2% ± 8.74% [85.6%, 90.8%]
Sensitivity	88.0% ± 11.9% [84.4%, 91.5%]	**88.2% ± 12.4%** **[84.5%, 91.8%]**	91.7% ± 9.21% [88.9%, 94.4%]	83.1% ± 14.8% [78.8%, 87.5%]
Specificity	92.2% ± 7.78% [89.9%, 94.5%]	**92.0% ± 8.00%** **[89.7%, 94.4%]**	85.2% ± 13.0% [81.3%, 89.0%]	91.1% ± 11.5% [87.7%, 94.5%]
Impedance VU‐AMS 5fs (*n* = 39)				
AUC	**97.0% ± 3.32%** **[95.9%, 98.0%]**	96.9% ± 3.62% [95.8%, 98.0%]	94.9% ± 5.15% [93.3%, 96.5%]	95.8% ± 5.21% [94.1%, 97.4%]
Accuracy	**90.8% ± 5.75%** **[88.9%, 92.6%]**	91.2% ± 5.11% [89.6%, 92.8%]	86.9% ± 8.16% [84.3%, 89.5%]	89.9% ± 6.11% [88.0%, 91.8%]
Sensitivity	**88.7% ± 10.2%** **[85.5%, 91.9%]**	90.3% ± 9.09% [87.4%, 93.1%]	89.6% ± 8.75% [86.9%, 92.4%]	84.3% ± 12.6% [80.4%, 88.2%]
Specificity	**91.9% ± 7.26%** **[89.7%, 94.2%]**	91.7% ± 6.84% [89.6%, 93.8%]	85.3% ± 11.7% [81.7%, 89.0%]	93.1% ± 6.82% [91.0%, 95.2%]
Impedance VU‐AMS Core (*n =* 38)				
AUC	**92.1% ± 8.93%** **[89.3%, 95.0%]**	91.6% ± 10.1% [88.4%, 94.9%]	90.2% ± 10.3% [87.0%, 93.5%]	90.6% ± 9.59% [87.5%, 93.6%]
Accuracy	**84.1% ± 10.3%** **[80.8%, 87.4%]**	84.3% ± 11.2% [80.8%, 87.9%]	81.3% ± 12.5% [77.3%, 85.2%]	83.6% ± 9.72% [80.5%, 86.7%]
Sensitivity	**79.3% ± 16.4%** **[74.1%, 84.5%]**	79.9% ± 17.0% [74.5%, 85.3%]	89.4% ± 9.23% [86.4%, 92.3%]	76.2% ± 16.3% [71.0%, 81.4%]
Specificity	**86.9% ± 12.0%** **[83.1%, 90.7%]**	87.0% ± 12.9% [82.8%, 91.1%]	76.7% ± 17.2% [71.2%, 82.1%]	87.9% ± 10.7% [84.5%, 91.3%]
Acc (*n* = 41)				
AUC	**99.3% ± 3.18%** **[98.3%, 100%]**	99.1% ± 3.30% [98.1%, 100%]	98.8% ± 4.18% [97.6%, 100%]	96.9% ± 6.51% [94.9%, 98.9%]
Accuracy	**97.6% ± 4.77%** **[96.1%, 99.1%]**	97.6% ± 4.93% [96.1%, 99.1%]	97.0% ± 5.66% [95.3%, 98.8%]	94.9% ± 7.97% [92.5%, 97.4%]
Sensitivity	**96.4% ± 8.26%** **[93.9%, 99.0%]**	96.5% ± 8.28% [94.0%, 99.1%]	95.9% ± 11.2% [92.5%, 99.4%]	88.7% ± 19.7% [82.7%, 94.7%]
Specificity	**98.3% ± 5.22%** **[96.7%, 99.9%]**	98.3% ± 5.35% [96.7%, 99.9%]	97.7% ± 5.89% [95.9%, 99.5%]	98.5% ± 6.11% [96.6%, 100%]

*Note:* Results of the best‐performing machine learning algorithm per method are printed in boldface.

As all methods' mean AUC was significantly higher than 70%, meaning their performance is at least acceptable, Hypothesis 1 is not rejected. In fact, all methods had higher than 90% AUC, and therefore their binary classification performance of speech segments can be considered excellent (Polo and Miot 2020).

### 3.8 Hypothesis 2: Is the 2‐Bands RIP Method the Superior Method for Speech Detection?

The 95% CI of 2‐bands RIP method's AUC, (95.4%, 97.8%), is not higher than and nonoverlapping with that of the other methods' AUC 95% CI, except for Method 3b (Impedance Core). Thus, Hypothesis 2 was rejected. However, the AUC (95% CI [98.3%, 100%]), accuracy (95% CI [96.1%, 99.1%]), sensitivity (95% CI [93.9%, 99.0%]), and specificity (95% CI [96.7%, 99.9%]) of the Acc method were all significantly higher than those of 2‐bands RIP and ImP (both devices) and also higher than that of the Thorax RIP (although the confidence intervals of their AUC and Sensitivity overlap). The Acc was therefore the superior method for speech detection.

### 3.9 Hypothesis 3: Does the Best Model of Each Method Predict Speech in an Out‐of‐Sample Dataset?

In order to train a best single model for each given method, its best‐performing algorithm identified in the previous steps was used with the set of hyperparameters that most frequently appeared in the outer loops of the nested cross‐validation. The values of the hyperparameters for each method can be found in the Supplementary (Section 3).

**FIGURE 6:**
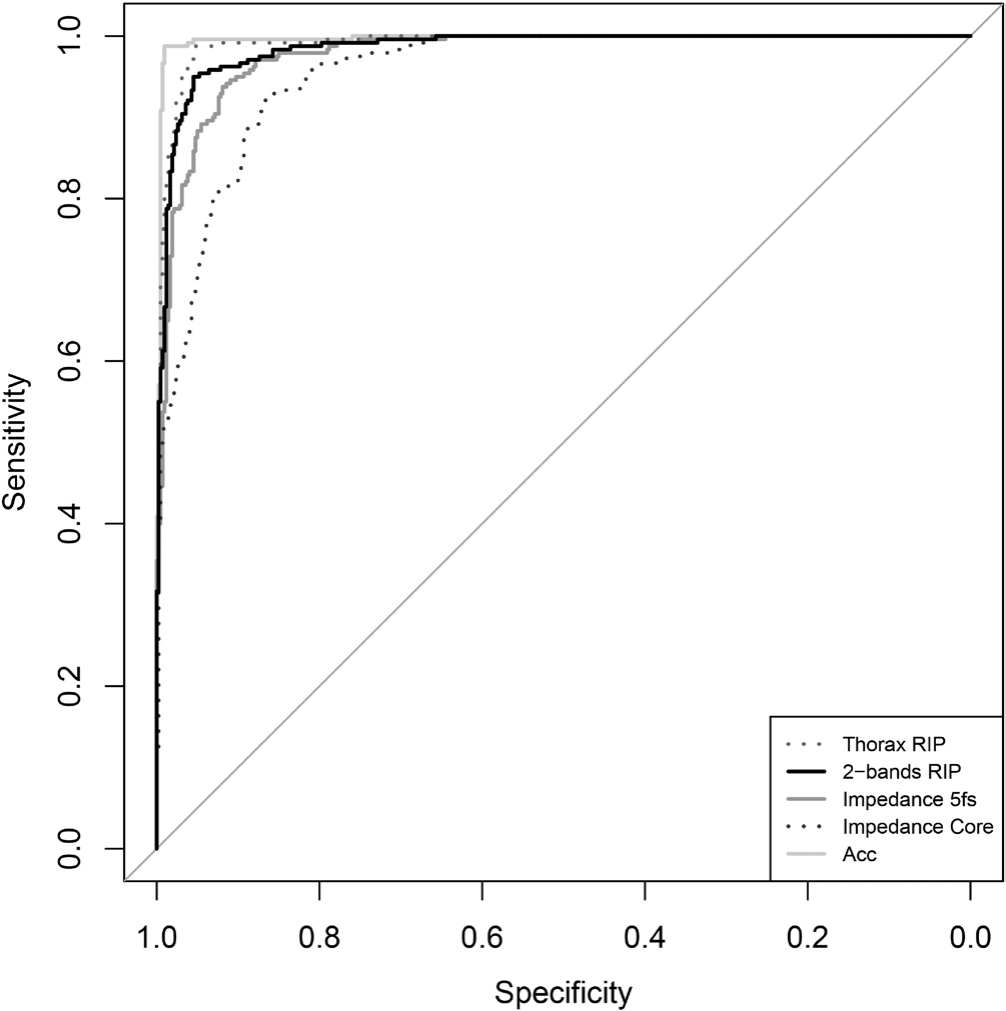
Receiver operating characteristics curve of each method's best model after predicting on the test set.

With the best single model of each method, predictions were made on the previously held‐out test set comprising of a total of 660 30‐s segments (*n* = 10). On this unseen test data, the best final model of all methods showed excellent speech detection performance. Figure 6 illustrates the receiver operating characteristics (ROC) curve per model as tested on the same test set. Thorax RIP (AUC = 99.1%, Accuracy = 95.9%, Sensitivity = 95.0%, Specificity = 96.4%), 2‐bands RIP (AUC = 98.5%, Accuracy = 94.8%, Sensitivity = 93.3%, Specificity = 95.7%), ImP 5fs (AUC = 97.8%, Accuracy = 92.0%, Sensitivity = 89.6%, Specificity = 93.3%), ImP Core (AUC = 95.7%, Accuracy = 87.3%, Sensitivity = 82.1%, Specificity = 90.2%), and Acc (AUC = 99.6%, Accuracy = 98.5%, Sensitivity = 98.8%, Specificity = 98.3%) all had an AUC higher than 70% in predicting the test set classifications. We, therefore, do not reject Hypothesis 3. Furthermore, as the performance indices did not noticeably decrease—but even increased—in the test set compared to those from the nested cross‐validation step for each method, there is no evidence of overfitting.

For the best model of each method, the feature importances were retrieved using the *feature_importances* function from scikit‐learn. It provides a metric for how much each feature is weighed by the model in making a prediction. Though the inspiration to expiration duration (IE ratio) has been emphasized in the past literature as a core feature in speech detection, we found that features capturing the variance in expiration duration (mean first difference of expiration and SD of expiratory duration) were the most important two features across respiratory methods. The top three features of the accelerometer (Acc) model were respectively root‐mean‐squared energy, spectral crest, and spectral entropy. Root‐mean‐square is a time‐domain feature reflecting the overall intensity of the signal. The higher energy of the voiced segments was indeed observed with the signal. Higher spectral crest and lower spectral entropy are indicative of peakiness of the spectrum, suggesting the voiced segments had more tonality and less white noise. See the Supplementary (Section 4, Supplementary Figures S1) for a visual representation of the feature importances for each model.

### 3.10 Hypothesis 4: Does the Best Model of 2‐Bands RIP Predict Speech Better Than Those of Other Methods?

Using the unseen test set, a DeLong's test between 2‐bands RIP and each of the other methods, including thorax RIP (*Z* = −2.69, *p* = 0.007), ImP 5fs (*Z* = 1.76, *p* = 0.079), ImP Core (*Z* = 4.83, *p* < 0.001), and Acc (*Z* = −3.00, *p* = 0.003) was run. Although the 2‐bands RIP performed significantly better than ImP Core, it did equally well as ImP 5fs, and significantly worse than Thorax RIP and Acc. Hypothesis 4 was therefore rejected. Of note, the Acc method significantly outperformed not only the ImP Core (*Z* = 6.13, *p* < 0.001) but also ImP 5 fs (*Z* = 4.28, *p* < 0.001) and 2‐bands RIP (*Z* = 3.00, *p* = 0.003). The Acc model, therefore, predicted speech better than the other methods.

### 3.11 Individual Differences in Speech Detection Performance

Our models were generic models, meaning they were based on data of all participants in the training set simultaneously. To assess whether the models performed well across individuals, we employed a two‐step approach. First, we used the data of the best nested cross‐validation in which every outer loop tested a model on the data of a different held‐out individual. Per method, histograms were created to illustrate the distribution of individual accuracy, AUC, sensitivity, and specificity percentages (see Supplementary, Section 9, Supplementary Figures S6–S10). For the 2‐bands RIP, Thorax RIP, impedance (VU‐AMS 5fs), and accelerometer methods, all AUC, accuracy, and specificity metrics at the individual level were always higher than 70%, except one instance of 69.7% accuracy for a participant in the Thorax RIP method. For all methods, sensitivity dropped below 70% for only a few individuals out of the approximately 40 outer loops (only one for the accelerometer method). Second, to assess whether model performance might be affected by biological sex, age, or body mass index (BMI), we correlated the AUC, accuracy, sensitivity, and specificity indices of the held‐out participants in the outer loops with their age, biological sex, and BMI. Models for impedance (VU‐AMS 5fs) had significantly higher specificity for females, indicating a better ability to correctly detect no‐speech periods, for females than they did for males, *N* = 39, Pearson's *r* = 0.357, *p* = 0.026. No other significant association was found between the performance metrics and age, sex, or BMI for any of the methods. Heatmaps of the correlation matrices can be found in the Supplementary (Section 9, Supplementary Figures S11–S15).

We additionally assessed how well each method's best (single) model generalized to the unseen test set across different subgroups. Performance metrics were calculated separately for males, females, low BMI, and high BMI groups (as determined by a median split of the overall sample; Median = 21.0 kg/m^2^) for making predictions on the test set. As the test set was mostly composed of young adults, we did not perform a median split for age. Sensitivity, defined as the ability to correctly detect speech periods, was similar across the entire sample and test set for the high BMI group. However, for all respiratory methods (thoracoabdominal RIP, thorax‐only RIP, and impedance), the low BMI group showed small decreases in sensitivity in the test set compared with the entire sample. For example, while the sensitivity for the 2‐bands RIP in the high BMI group only slightly decreased from 97.5% (entire sample) to 95.8% (in the test set), sensitivity for the low BMI group decreased from 96.2% to 89.6%, though it remained well above acceptable levels. No such drop in sensitivity for the low BMI group occurred when using the accelerometer method. For the tables of the performance metrics for these subgroups as tested on both the entire sample and only the test set, see the Supplementary (Section 9, Supplementary Tables S15–S18).

### 3.12 Performance of the Accelerometer Method at 3‐s Segments

To explore whether the best‐performing method of sternal accelerometry would retain its performance while detecting speech in shorter time segments, we used the existing data to extract the exact same 10 features, this time for every 3 seconds. In total, 27,060 3‐s segments (660 per person) were obtained for the training set, and 6600 obtained for the test set. A model was subsequently trained using the previously determined learning algorithm (Gradient Boosting) and set of hyperparameters. While tested on the test set, excellent levels of AUC (98.8%), accuracy (97.5%), sensitivity (96.5%), and specificity (98.1%) were maintained.

## 4 Discussion

Speech activity obscures the true interrelationships between psychosocial exposures and physiological parameters including heart rate, heart rate variability, skin conductance level, nonspecific skin conductance responses, blood pressure, and T‐wave amplitude (Arnold et al. 2014; Bernardi et al. 2000; Brugnera et al. 2018; Friedmann et al. 1982; Liehr 1992; Linden 1987; Linden and Estrin 1988; Lynch et al. 1980; Quintana and Heathers 2014; Reilly and Moore 2003; Sloan et al. 1991; Strauss‐Blasche et al. 2000; Weber and Smith 1990). In naturalistic settings, a major obstacle is detecting when speech occurs in a privacy‐sensitive manner. Fortunately, a number of biosignals have differing characteristics while speaking or not speaking, enabling us to develop viable methods for speech detection without compromising participant privacy.

In a controlled laboratory study, we used machine learning to train, comprehensively test, and statistically compare between the speech detection performances of respiratory inductance plethysmography (RIP) with both thorax and abdomen respiration, RIP with only thorax respiration, impedance pneumography, and an upper sternum (near‐larynx) placed accelerometer‐gyroscope unit. To provide guidance for future researchers seeking to take speech production into account when interpreting ambulatory physiology, we solely utilized noninvasive research‐oriented wearable monitors for these biosignals. All biosignals showed an excellent ability to differentiate between speech and no‐speech periods, reflected in the areas under the curve of higher than 90%. This was true for both their nested cross‐validation (nCV) within the training set and for the application of the best model in the test set. This suggests the models of these methods would perform well in a larger sample when the positive biases are minimized. In the nCV, a *collection* of models is essentially created per method to be able to do this testing repeatedly for all participants in the training set. Apart from providing a proof‐of‐concept for speech detection from physiological signals, a best model was trained per method—using the best‐performing machine learning algorithm (e.g., XGBoost) and the most frequently occurring hyperparameters. Since the performance indices of the best models as tested on the test set did not decrease compared to those in the nCV, there was no apparent evidence of overfitting in the current study. Also, while the models were trained using data from all participants simultaneously, they held well at the level of a single individual, with AUC's rarely lower than 70%.

In contrast to our expectation, using both the thoracic and abdominal respiratory belts for speech detection did not increase any of AUC, accuracy, sensitivity, or specificity over a thorax‐only RIP model. Here, using only the thorax band will not just reduce the data processing effort but will also enhance the accuracy of speech detection. Nevertheless, in some research scenarios, such as studying respiratory changes in response to emotion and arousal, recording both thoracic and abdominal breathing could be crucial (Boiten et al. 1994). Impedance pneumography performed as well as thoracoabdominal (2‐bands) RIP in detecting speech. Notably, it was the sternal accelerometer that outperformed the other methods, with regards to all metrics used, that is, accuracy, AUC, sensitivity, and specificity. Sternal accelerometers require significantly less processing effort as an intermediary peak‐trough identification step (unlike the respiratory methods) is not needed. Sternal accelerometers can be implemented with relatively lower burden for the participant, and might therefore facilitate detection of speech episodes for studies of longer duration. The accelerometers, upon lowpass filtering to eliminate speech‐related signal content and calculation of vector magnitude, can also be used to control for the effects of physical activity and posture (Van De Ven et al. 2024) on physiological parameters, achieving three goals at once. After examining whether model performances for the different methods might decrease due to certain individual characteristics, we found no impact of age or sex on model performances. However, the models using impedance pneumography and respiratory inductance plethysmography data generalized slightly less well (with regard to sensitivity) to individuals with lower body mass index. Sternal accelerometers did not show any decrease in performance based on age, biological sex, or body mass index and may have relatively better external validity.

An online open‐access repository (https://github.com/melisasaygin/SpeechDetection) was created, containing the trained models with the associated scripts (e.g., feature extraction per method). In the Supplementary Sections 5 and 6, a walkthrough of the steps to take for each method is provided. This should allow researchers to apply the models, validated with laboratory tasks simulating different types of speech, to new data collected using any of thorax/abdominal respiration, across thorax impedance, or upper sternal accelerometers (see Supplementary, Section 7 for sensor specifications). For interested readers, Section 7 of the Supplementary discusses some example research scenarios for each of the speech detection methods in the current paper.

The major application of speech detection by these methods is to allow a better interpretation of co‐recorded physiological (i.e., autonomic) measures. Biosignal‐based speech detection could be used in future ambulatory studies to stratify analyses by speaking and nonspeaking periods. Alternatively, speaking can be added as a covariate to models (e.g., multilevel regression) linking dynamic psychological states (e.g., stress, positive affect) to physiological responses. A primary example is a scenario where the reduction in heart rate variability under stress is masked during active speaking. Stratified analysis would uncover such masking.

Other use cases of speech detection may be the tracking of social interactive behaviors of those with higher social anxiety levels or hearing impairment. In individuals with social anxiety disorder, the initiation or maintenance of conversation and public speaking are among the most stress‐inducing situations (Lecrubier et al. 2000). It should be noted that a common vocal characteristic in social anxiety disorder is a higher pitch of the fundamental frequency (Weeks et al. 2012). As the models (including that for accelerometer) do not depend on a particular fundamental frequency range, they are likely to perform equally well within these populations. Subvocal speech may also be observed in social anxiety, in which case the respiratory methods, relying on the variability of expiratory durations instead of speech volume, would likely show good generalizability. In those with hearing impairments, conversation elicits heightened listening effort and, consequently, stress (Shields et al. 2023). The passive identification of when these individuals spoke can be a convenient and low‐burden way of identifying potential acute stress periods without entirely depending on self‐report (Boukhechba et al. 2018; Ernst et al. 2024). It was also suggested that the amount of own speech may act as an approximate index of time spent alone (Ejupi and Menon 2018) and might capture the increased social isolation that is caused by hearing impairment (Shukla et al. 2020).

### 4.1 Limitations and Future Directions

Although methodologically robust and inclusive of different speech types that would be typically produced in daily life, the validation results for the methods described in this paper cannot be generalized to settings outside of a controlled laboratory experiment without further work. Performance of the models should now be established in prolonged ambulatory recordings where a ground truth for speech is also available. This could involve comparison to continuous speech recording using a throat microphone, while taking appropriate measures to safeguard participant and bystander privacy.

A second limitation is that we did not calibrate the respiratory signals against a spirometer to obtain absolute volume of respiration, despite that the mean inspiratory and expiratory respiration amplitude features were not directly used in the models as they were not among the top 10 selected features. Although a manipulation check was performed in the physiological sense by comparing heart rate due to condition (e.g., serial subtraction) and speech presence separately, participants were not asked to report their level of perceived stress following the conditions.

A further limitation of the current study is that we trained the models with speech segments filled with continuous speech (i.e., 30 s of conversation *including* typical pauses in speech). In daily life participants may speak for shorter intervals than 30 s, take longer pauses, or a single speech episode may be split among multiple windows. From the current experiment, it is not clear what proportion of speech would need to be in a 30 s window for these models to classify it as a speech segment. To get some idea of this, we used the only method that allowed to extract the core features even in very short 3‐s fragments, the accelerometer method. Results were reassuring and showed that the accelerometer method maintained excellent performance in differentiation between speech and no‐speech segments. However, it is unclear how this would be for the other three methods. Therefore, future research should examine the behavior of the models at lower proportions of speech per segment. Ideally, further research should also include a larger and more diverse sample with regard to gender balance, age, and health conditions, to better establish model generalizability. Since the current study's sample was predominantly composed of young adults and had a gender imbalance, future studies should reassess how well the speech detection models generalize to both male and older participants.

### 4.2 Conclusion

Speech production is a primary means of human communication. It has unique respiratory demands since the voice needs to be carried on the prolonged expiratory phase of a breath. Numerous physiological parameters commonly utilized in psychophysiological research such as heart rate, heart rate variability, blood pressure, and skin conductance response change along with the respiratory changes induced by speech. If not accounted for, these changes in parameters may mistakenly be attributed to psychological constructs of interest, exaggerating or concealing true psychophysiological linkages. Though physical activity and posture are acknowledged as confounding factors in ambulatory research, detecting, and controlling for speech has not been common practice. Notably, in the current study, speaking not only had a stronger effect on heart rate than the mental stressor of interest, but also than the postural changes typically considered the more critical confounder. Using wearable monitors that were all amenable to ambulatory recordings, we trained, validated, and compared machine learning models for speech detection for respiratory inductance plethysmography, thorax impedance, and a triaxial accelerometer unit positioned at upper sternum. While all models demonstrated excellent performance across various metrics such as area under the curve, accuracy, sensitivity, and specificity; the sternal accelerometers generally outperformed the other methods in all performance metrics. We expect the validity of these models to hold in daily life settings. If so, they can be used to stratify psychophysiological analysis of ambulatory data based on speech presence, or statistically control for the effects of speech on the physiological variables recorded.

## Author Contributions

**Melisa Saygin:** conceptualization, data curation, formal analysis, investigation, methodology, resources, validation, visualization, writing – original draft, writing – review and editing. **Myrte Schoenmakers:** conceptualization, data curation, investigation, methodology, resources, writing – review and editing. **Martin Gevonden:** conceptualization, formal analysis, methodology, visualization, writing – review and editing. **Eco de Geus:** conceptualization, formal analysis, funding acquisition, methodology, visualization, writing – review and editing.

## Conflicts of Interest

The authors declare no conflicts of interest.

## Supporting information


Data S1.

## Data Availability

All processed (feature) data is available on the open‐access GitHub repository (https://github.com/melisasaygin/SpeechDetection). Raw data will be made available upon reasonable request to the corresponding author. Example raw (signal) data of a participant who provided consent for their raw data to be open access is included in the repository.
