# Supplementary material for: Speech Detection via Respiratory Inductance Plethysmography, Thoracic Impedance, Accelerometers, and Gyroscopes: A Machine Learning‐Informed Comparative Study
Source: Psychophysiology. 2025 Feb 14;62(2):e70021. doi: 10.1111/psyp.70021 (PMC11826986; doi:10.1111/psyp.70021)
Supplement: Supplementary file 1 — Data S1. [file PSYP-62-e70021-s001.docx]

**Supplementary to** **Speech Detection via Respiratory Inductance Plethysmography, Thoracic Impedance, Accelerometers and Gyroscopes: A Machine Learning-Informed Comparative Study**

Melisa Saygin ^1,2^, Myrte Schoenmakers ^1,2^, Martin Gevonden ^1,2^, Eco de Geus ^1,2^

^1^ Department of Biological Psychology, VU Amsterdam, Amsterdam, The Netherlands

^2^ Amsterdam Public Health research institute, Amsterdam UMC, Amsterdam, The Netherlands

Contents

[1. Effects of speech, posture, condition, and method on respiratory rate and IE-ratio 2](#_Toc182842988)

[2. [The ten most informative features of each method as determined by information gains 7](#_Toc182842989)](#_Toc182842990)

[3. Hyperparameters of the best model of each method 10](#_Toc182842991)

[4. Feature importances for the best final model of each method 10](#_Toc182842992)

[5. Description of the structure of the GitHub repository with scripts and example datasets 16](#_Toc182842993)

[6. Instructions to use the methods for speech detection 17](#_Toc182842994)

[6.1 Speech detection using respiratory inductance plethysmography 17](#_Toc182842995)

[6.2 Speech detection using impedance pneumography 18](#_Toc182842996)

[6.3 Speech detection using accelerometers on sternum 19](#_Toc182842997)

[7. Potential use of the methods for speech detection 19](#_Toc182842998)

[7.1 Speech detection using respiratory inductance plethysmography 19](#_Toc182842999)

[7.2 Speech detection using impedance pneumography 20](#_Toc182843000)

[7.3 Speech detection using accelerometers on sternum 21](#_Toc182843001)

[8. Rationale for bypassing the feature normalization step 21](#_Toc182843002)

[9. Individual differences in model performance: AUC, Accuracy, Sensitivity, Specificity 23](#_Toc182843003)

# Effects of speech, posture, condition, and method on respiratory rate and IE-ratio

For the conditions of reading and conversation, two Greenhouse-Geisser (GG) adjusted four-way 4 (respiration signal type) x 3 (posture) x 2 (condition) x 2 (speech presence) repeated measures (rm) ANOVAs were run: one for respiration rate (RR) and the other for inspiration to expiration ratio (IE-ratio) as the outcome variable. Significance threshold was set to .005. All factors except respiration signal type, *p* = .745, had a significant main effect on RR: speech presence (*M*_silent_ = 17.4, *M*_aloud_ = 14.5), *F*_1, 43_ = 55.8, *p* < .001, η_p_^2^ = .565; posture (*M*_supine_ = 16.5, *M*_sitting_ = 15.5, *M*_standing_ = 15.9), *F*_1.69, 72.6_ = 16.6, *p* < .001, η_p_^2^ = .278; experimental condition (*M*_reading_ = 16.3, *M*_conversation_= 15.6), *F*_1, 43_ = 15.2, *p* < .001, η_p_^2^ = .261. Both speech presence (*M*_silent_ = .881, *M*_aloud_ = .721), *F*_1, 43_ = 108, *p* < .001, η_p_^2^ = .716, and the signal type used (*M*_thoraxRIP_ = .757, *M*_abdomenRIP_ = .748, *M*_ImP5fs_ = .820, *M*_ImPCore_ = .879), *F*_2.37, 102_ = 18.6, *p* < .001, η_p_^2^ = .301, had significant main effects on IE-ratio while posture (*p* = .024) and experimental condition (*p* = .741) did not.

For the conditions of reading while standing and walking, the three-way 4 x (respiration signal type) x 2 (condition) x 2 (speech presence) rm-ANOVA showed that respiration signal type (*M*_thoraxRIP_ = 18.4, *M*_abdomenRIP_ = 18.5, *M*_ImP5fs_ = 18.1, *M*_ImPCore_ = 17.7), *F*_2.43, 105_ = 12.6, *p* < .001, η_p_^2^ = .226, condition (*M*_reading_ = 16.2, *M*_walking_= 20.1), *F*_1.00, 43.0_ = 291, *p* < .001, η_p_^2^ = .871, and speech presence (*M*_silent_ = 21.3, *M*_aloud_ = 15.1), *F*_1.00, 43.0_ = 242, *p* < .001, η_p_^2^ = .849 all had a significant effect on respiratory rate. The ANOVA was repeated when IE-ratio is the outcome variable. While the experimental condition (*M*_reading_ = .816, *M*_walking_ = .891), *F*_1.00, 43.0_ = 21.9, *p* < .001, η_p_^2^ = .338 and speech presence (*M*_silent_ = .941, *M*_aloud_ = .766), *F*_1.00, 43.0_ = 68.0, *p* < .001, η_p_^2^ = .613, had a significant main effect, signal type did not, *p =* .008.

Using the conditions of reading while sitting and serial subtraction, the three-way 4 x (respiration signal type) x 2 (condition) x 2 (speech presence) rm-ANOVA with RR as the outcome variable showed that condition (*M*_reading_ = 15.8, *M*_serialsubtract_ = 17.0), *F*_1.00, 43.0_ = 18.6, *p* < .001, η_p_^2^ = .302, and speech presence (*M*_silent_ = 17.8, *M*_aloud_ = 15.0), *F*_1.00, 43.0_ = 36.5, *p* < .001, η_p_^2^ = .459 had a significant main effect, while respiration signal type did not, *p* = .092. The rm-ANOVA was repeated with IE-ratio as the outcome variable, and while speech presence (*M*_silent_ = .849, *M*_aloud_ = .737), *F*_1.00, 43.0_ = 33.7, *p* < .001, η_p_^2^ = .440, and signal type (*M*_thoraxRIP_ = .764, *M*_abdomenRIP_ = .740, *M*_ImP5fs_ = .820, *M*_ImPCore_ = .849), *F*_2.17, 93.1_ = 6.85, *p* = .001, η_p_^2^ = .137, had a significant main effect on IE-ratio, condition did not, *p =* .807.

**Supplementary Table 1**

*Four-way repeated measures ANOVA with respiratory rate as the dependent variable, in which condition levels are reading and conversation*

|  | *df* | *F* | *Sig.* | η_p_^2^ |
| --- | --- | --- | --- | --- |
| Signal | 2.42, 104 | .351 | .745 | .008 |
| Posture | 1.69, 72.6 | 16.6 | <.001 | .278 |
| Condition | 1.00, 43.0 | 15.2 | <.001 | .261 |
| Speech | 1.00, 43.0 | 55.8 | <.001 | .565 |
| Signal X Posture | 4.32, 182 | 1.61 | .169 | .036 |
| Signal X Condition | 2.49, 107 | 6.47 | .001 | .131 |
| Posture X Condition | 1.81, 77.8 | .066 | .921 | .002 |
| Signal X Posture X Condition | 4.63, 199 | 1.08 | .373 | .024 |
| Signal X Speech | 2.57, 110 | 1.13 | .335 | .026 |
| Posture X Speech | 1.89, 81.5 | 6.66 | .003 | .134 |
| Signal X Posture X Speech | 4.96, 213 | 4.22 | .001 | .089 |
| Condition X Speech | 1.00, 43.0 | 29.7 | <.001 | .408 |
| Signal X Condition X Speech | 2.53, 109 | 3.13 | .036 | .068 |
| Posture X Condition X Speech | 1.87, 80.3 | .179 | .822 | .004 |
| Signal X Posture X Condition X Speech | 5.26, 226 | .660 | .662 | .015 |

**Supplementary Table 2**

*Four-way repeated measures ANOVA with IE-ratio as the dependent variable, in which condition levels are reading and conversation*

|  | *df* | *F* | *Sig.* | η_p_^2^ |
| --- | --- | --- | --- | --- |
| Signal | 2.37, 102 | 18.6 | <.001 | .301 |
| Posture | 1.80, 77.6 | 4.07 | .024 | .087 |
| Condition | 1.00, 43.0 | .111 | .741 | .003 |
| Speech | 1.00, 43.0 | 108 | <.001 | .716 |
| Signal X Posture | 4.66, 201 | 2.12 | .069 | .047 |
| Signal X Condition | 2.48, 107 | 3.10 | .038 | .067 |
| Posture X Condition | 1.70, 72.9 | 1.34 | .266 | .030 |
| Signal X Posture X Condition | 3.98, 171 | 1.33 | .261 | .030 |
| Signal X Speech | 2.65, 114 | 10.8 | <.001 | .200 |
| Posture X Speech | 1.93, 83.1 | 11.5 | <.001 | .211 |
| Signal X Posture X Speech | 4.56, 196 | 1.12 | .351 | .025 |
| Condition X Speech | 1.00, 43.0 | 2.15 | .150 | .048 |
| Signal X Condition X Speech | 2.57, 110 | .968 | .400 | .022 |
| Posture X Condition X Speech | 1.98, 85.0 | .390 | .676 | .009 |
| Signal X Posture X Condition X Speech | 4.50, 194 | 1.57 | .178 | .035 |

**Supplementary Table 3**

*Three-way repeated measures ANOVA with respiratory rate as the dependent variable, in which condition levels are reading while standing and walking*

|  | *df* | *F* | *Sig.* | η_p_^2^ |
| --- | --- | --- | --- | --- |
| Signal | 2.43, 105 | 12.6 | <.001 | .226 |
| Condition | 1.00, 43.0 | 291 | <.001 | .871 |
| Speech | 1.00, 43.0 | 242 | <.001 | .849 |
| Signal X Condition | 2.01, 86.4 | .734 | .483 | .017 |
| Signal X Speech | 2.18, 93.9 | 6.60 | .002 | .133 |
| Condition X Speech | 1.00, 43.0 | 97.4 | <.001 | .694 |
| Signal X Condition X Speech | 2.00, 85.9 | 5.09 | .008 | .106 |

**Supplementary Table 4**

*Three-way repeated measures ANOVA with IE-ratio as the dependent variable, in which condition levels are reading while standing and walking*

|  | *df* | *F* | *Sig.* | η_p_^2^ |
| --- | --- | --- | --- | --- |
| Signal | 2.70, 116 | 4.32 | .008 | .091 |
| Condition | 1.00, 43.0 | 21.9 | <.001 | .338 |
| Speech | 1.00, 43.0 | 68.0 | <.001 | .613 |
| Signal X Condition | 2.90, 125 | 3.36 | .022 | .072 |
| Signal X Speech | 2.94, 126 | 5.28 | .002 | .109 |
| Condition X Speech | 1.00, 43.0 | 5.86 | .020 | .120 |
| Signal X Condition X Speech | 2.60, 112 | .923 | .421 | .021 |

**Supplementary Table 5**

*Three-way repeated measures ANOVA with respiratory rate as the dependent variable, in which condition levels are reading while sitting and serial subtraction*

|  | *df* | *F* | *Sig.* | η_p_^2^ |
| --- | --- | --- | --- | --- |
| Signal | 2.04, 87.8 | 2.44 | .092 | .054 |
| Condition | 1.00, 43.0 | 18.6 | <.001 | .302 |
| Speech | 1.00, 43.0 | 36.5 | <.001 | .459 |
| Signal X Condition | 2.78, 120 | .287 | .820 | .007 |
| Signal X Speech | 2.53, 109 | .036 | .983 | .001 |
| Condition X Speech | 1.00, 43.0 | .152 | .698 | .004 |
| Signal X Condition X Speech | 2.65, 114 | .090 | .953 | .002 |

**Supplementary Table 6**

*Three-way repeated measures ANOVA with IE-ratio as the dependent variable, in which condition levels are reading while sitting and serial subtraction*

|  | *df* | *F* | *Sig.* | η_p_^2^ |
| --- | --- | --- | --- | --- |
| Signal | 2.17, 93.1 | 6.85 | .001 | .137 |
| Condition | 1.00, 43.0 | .061 | .807 | .001 |
| Speech | 1.00, 43.0 | 33.7 | <.001 | .440 |
| Signal X Condition | 2.35, 101 | .396 | .707 | .009 |
| Signal X Speech | 2.65, 114 | 3.83 | .015 | .082 |
| Condition X Speech | 1.00, 43.0 | 14.8 | <.001 | .256 |
| Signal X Condition X Speech | 2.65, 114 | .533 | .638 | .012 |

**Supplementary Table 7**

Three-way repeated measures ANOVA with heart rate as the dependent variable

|  | *df* | *F* | *Sig.* | η_p_^2^ |
| --- | --- | --- | --- | --- |
| Posture | 1.23, 59.2 | 115 | <.001 | .705 |
| Condition | 1.00, 48.0 | 15.7 | <.001 | .247 |
| Speech | 1.00, 48.0 | 226 | <.001 | .825 |
| Posture X Condition | 1.98, 94.8 | 9.97 | <.001 | .172 |
| Posture X Speech | 1.84, 88.4 | 20.8 | <.001 | .302 |
| Condition X Speech | 1.00, 48.0 | 85.5 | <.001 | .641 |
| Posture X Condition X Speech | 1.96, 94.2 | 3.83 | .026 | .074 |

*Note*. Condition levels included in the above rm-ANOVA are reading and conversation.

**Supplementary Table 8**

Two-way repeated measures ANOVA with heart rate as the dependent variable

|  | *df* | *F* | *Sig.* | η_p_^2^ |
| --- | --- | --- | --- | --- |
| Condition | 1.00, 48.0 | 75.7 | <.001 | .612 |
| Speech | 1.00, 48.0 | 15.9 | <.001 | .249 |
| Condition X Speech | 1.00, 48.0 | 2.48 | .122 | .049 |

*Note*. Condition levels included in the above rm-ANOVA are reading while standing and walking.

**Supplementary Table 9**

Two-way repeated measures ANOVA with heart rate as the dependent variable

|  | *df* | *F* | *Sig.* | η_p_^2^ |
| --- | --- | --- | --- | --- |
| Condition | 1.00, 48.0 | 14.8 | <.001 | .236 |
| Speech | 1.00, 48.0 | 200 | <.001 | .806 |
| Condition X Speech | 1.00, 48.0 | 29.4 | <.001 | .380 |

*Note*. Condition levels included in the above rm-ANOVA are reading while sitting and serial subtraction.

# The ten most informative features of each method as determined by information gains

Below are the top ten features that were selected for each method. This was done using the *mutual_info_classif* function from *scikit-learn* on the training set data only, which provides an information gain statistic based on how much a feature minimizes the entropy in the target variable (speech – no speech). For the machine learning pipeline, including both the stages of nested cross-validation and testing of the best model on the test set, only the below specified ten features were used for the models of a given method.

Along with the selected ten features, the tables below also include the information gain statistic per feature, and the variable name used for a given feature in our scripts.

**Supplementary Table 10**

*The ten features selected and used for the Thorax RIP method, with level of information gain*

| Feature | | Variable Name | Information Gain |
| --- | --- | --- | --- |
| Mean first difference of expiration | *exp_diff_mean* | | .306 |
| SD of expiratory duration | *exp_dur_sd* | | .291 |
| Mean duty cycle | *duty_cycle_mean* | | .274 |
| Respiratory rate variability | *RRV_RMSSD* | | .255 |
| Mean IE-ratio | *ie_ratio_mean* | | .204 |
| SD of inspiratory flow rate | *insp_flow_sd* | | .186 |
| SD of expiratory amplitude | *exp_amp_sd* | | .146 |
| SD of inspiratory amplitude | *insp_amp_sd* | | .144 |
| SD of IE-ratio | *ie_ratio_sd* | | .126 |
| Mean expiratory duration | *exp_dur_mean* | | .122 |

**Supplementary Table 11**

*The ten features selected and used for the 2-bands RIP method, with level of information gain*

| Feature | Variable Name | Information Gain |
| --- | --- | --- |
| SD of expiratory duration (abdomen) | *A_exp_dur_sd* | .311 |
| Mean first difference of expiration (thorax) | *exp_diff_mean* | .306 |
| Mean first difference of expiration (abdomen) | *A_exp_diff_mean* | .305 |
| SD of expiratory duration (thorax) | *exp_dur_sd* | .291 |
| Mean duty cycle (thorax) | *duty_cycle_mean* | .274 |
| Respiratory rate variability (abdomen) | *A_RRV_RMSSD* | .261 |
| Respiratory rate variability (thorax) | *RRV_RMSSD* | .255 |
| Mean IE-ratio (thorax) | *ie_ratio_mean* | .204 |
| Mean duty cycle (abdomen) | *A_duty_cycle_mean* | .199 |
| SD of inspiratory flow rate (thorax) | *insp_flow_sd* | .186 |

**Supplementary Table 12**

*The ten features selected and used for the Impedance (5fs) method, with level of information gain*

| Feature | Variable Name | Information Gain |
| --- | --- | --- |
| Mean first difference of expiration | *ImP_exp_diff_mean* | .293 |
| SD of expiratory duration | *ImP_exp_dur_sd* | .282 |
| Respiratory rate variability | *ImP_RRV_RMSSD* | .254 |
| SD of inspiratory flow rate | *ImP_insp_flow_sd* | .184 |
| SD of IE-ratio | *ImP_ie_ratio_sd* | .180 |
| Mean duty cycle | *ImP_duty_cycle_mean* | .158 |
| SD of inspiratory amplitude | *ImP_insp_amp_sd* | .142 |
| Peak-trough symmetry | *ImP_RSP_Symmetry_PeakTrough* | .125 |
| SD of expiratory amplitude | *ImP_exp_amp_sd* | .117 |
| Mean expiratory duration | *ImP_exp_dur_mean* | .104 |

**Supplementary Table 13**

*The ten features selected and used for the Impedance (Core) method, with level of information gain*

| Feature | Variable Name | Information Gain |
| --- | --- | --- |
| SD of expiratory duration | *Pro_exp_dur_sd* | .245 |
| Mean first difference of expiration | *Pro_exp_diff_mean* | .240 |
| Respiratory rate variability | *Pro_RRV_RMSSD* | .211 |
| SD of IE-ratio | *Pro_ie_ratio_sd* | .117 |
| Mean duty cycle | *Pro_duty_cycle_mean* | .116 |
| Mean expiratory duration | *Pro_exp_dur_mean* | .086 |
| Mean IE-ratio | *Pro_ie_ratio_mean* | .075 |
| SD of inspiratory flow rate | *Pro_insp_flow_sd* | .073 |
| SD of inspiratory duration | *Pro_insp_dur_sd* | .067 |
| Respiratory rate | *Pro_RSP_Rate_Mean* | .064 |

**Supplementary Table 14**

*The ten features selected and used for the Accelerometer method, with level of information gain*

| Feature | Variable Name | Information Gain |
| --- | --- | --- |
| Spectral variance (sagittal) | *AccZ_Spectral_Variance* | .518 |
| Root-mean-squared (sagittal) | *AccZ_RMS* | .516 |
| Spectral skewness (sagittal) | *AccZ_Spectral_Skewness* | .503 |
| Spectral mean (sagittal) | *AccZ_Spectral_Mean* | .492 |
| Spectral kurtosis (sagittal) | *AccZ_Spectral_Kurtosis* | .482 |
| Spectral flatness (sagittal) | *AccZ_Spectral_Flatness* | .478 |
| Spectral entropy (sagittal) | *AccZ_Spectral_Entropy* | .462 |
| Spectral crest (sagittal) | *AccZ_Spectral_Crest* | .445 |
| Zero crossing rate (sagittal) | *AccZ_ZCR* | .414 |
| 50% Spectral Rolloff (sagittal) | *AccZ_Spectral_Rolloff_50* | .384 |

# Hyperparameters of the best model of each method

For the XGBoost based classifier of the thorax RIP method, the following were entered as hyperparameters: *learning_rate* = .1, *n_estimators* = 200, *max_depth* = 3, *subsample* = .9, *min_child_weight* = 1, *gamma* = 0, and *colsample_bytree* = .8.

For the XGBoost based classifier of 2-bands (thoracoabdominal) RIP, hyperparameters entered were *learning_rate* = .1, *n_estimators* = 200, *max_depth* = 4, *subsample* = .9, *min_child_weight* = 2, *gamma* = 1, and *colsample_bytree* = 1.

For the Impedance method (as measured by VU-AMS 5fs), the hyperparameters of the Gradient Boosting algorithm were *learning_rate* = .1, *n_estimators* = 200, *max_depth* = 4, *subsample* = .9, *max_features* = ‘sqrt’, *min_samples_leaf* = 1, *min_samples_split* = 2, .

For the Impedance method (as measured by VU-AMS Core), the Gradient Boosting based classifier’s hyperparameters were *learning_rate* = .1, *n_estimators* = 100, *max_depth* = 3, *subsample* = 1, *max_features* = ‘sqrt’, *min_samples_leaf* = 1, *min_samples_split* = 2

For the Gradient Boosting classifier of the accelerometer (Acc) method, hyperparameters were as follows: *learning_rate* = .1, *n_estimators* = 200, *max_depth* = 4, *subsample* = 1, *max_features* = ‘sqrt’, *min_samples_leaf* = 1, *min_samples_split* = 2.

For a definition on what each hyperparameter refers to, see <https://scikit-learn.org/dev/modules/generated/sklearn.ensemble.GradientBoostingClassifier.html> for the Gradient Boosting classifier hyperparameters, and see [https://xgboost.readthedocs.io/en/sSupplementary Table/parameter.html#parameters-for-tree-booster](https://xgboost.readthedocs.io/en/stable/parameter.html#parameters-for-tree-booster) for the XGBoost classifier hyperparameters.

# Feature importances for the best final model of each method

Below, for each method's best single trained model, there is a bar plot showing feature importances for all the features used by a model. Feature importance quantifies how much a model would weigh a feature while making a speech-no speech prediction. The feature importance values were obtained using the *feature_importances* function of *scikit-learn*. Feature importance is a model-specific property and will remain consistent once a model has been trained.

**Supplementary Figure 1**

*Graph of the importance of each feature for the Thorax RIP model*


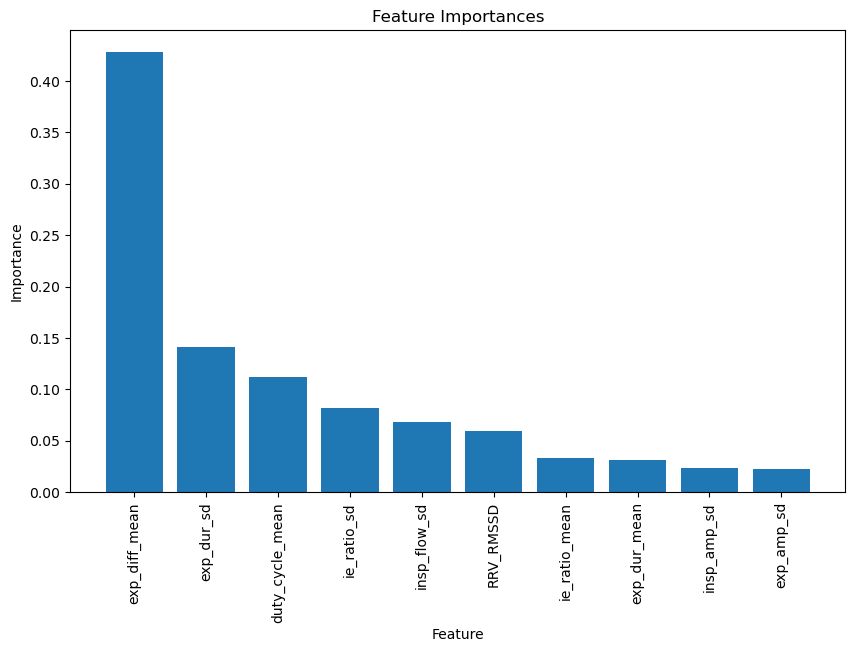


**Supplementary Figure 2**

*Graph of the importance of each feature for the 2-bands (thoracoabdominal) RIP model*


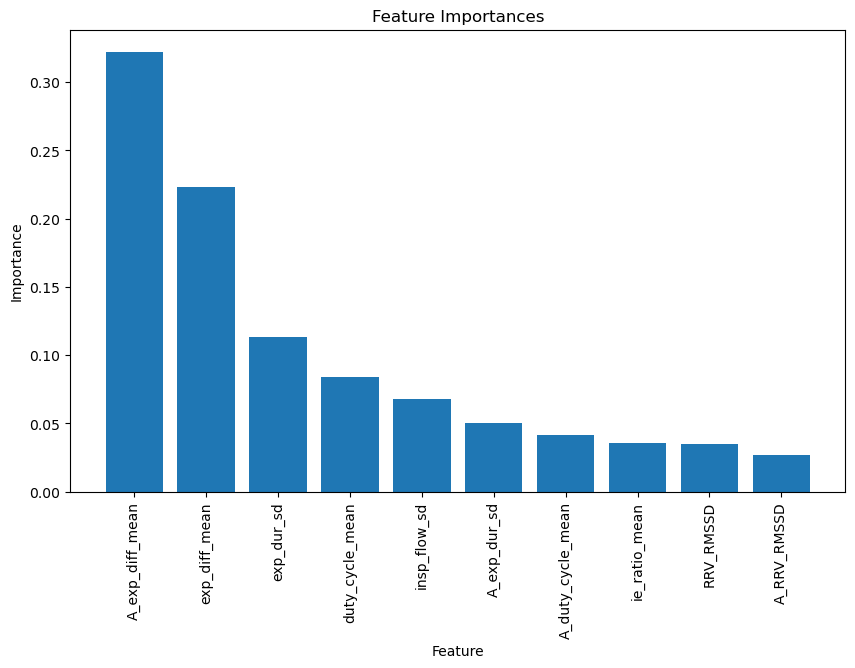


**Supplementary Figure 3**

*Graph of the importance of each feature for the Impedance model (with VU-AMS 5fs)*


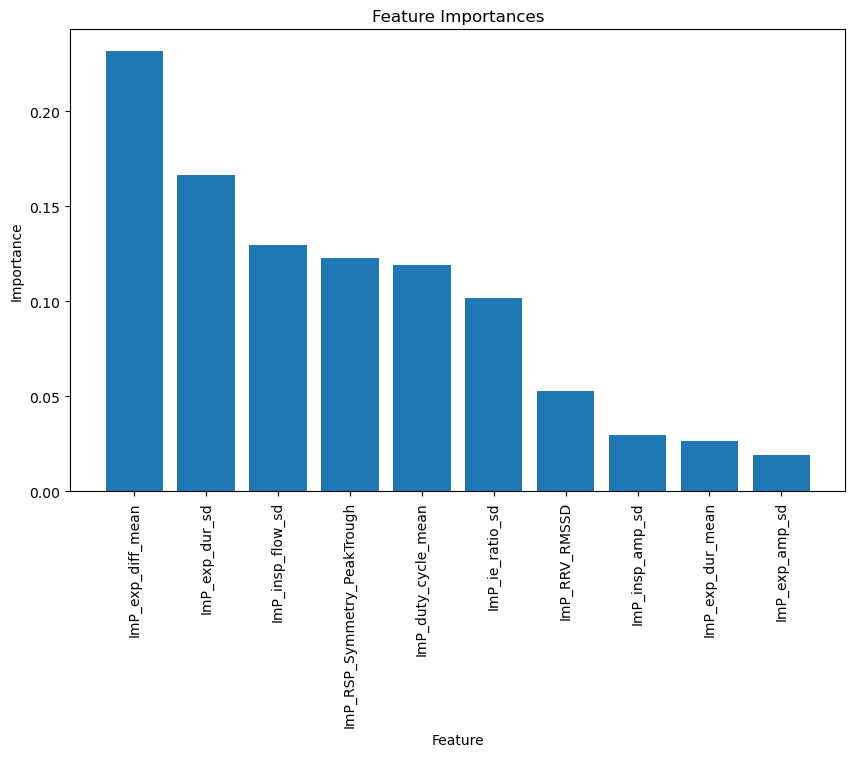


**Supplementary Figure 4**

*Graph of the importance of each feature for the Impedance model (with VU-AMS Core)*


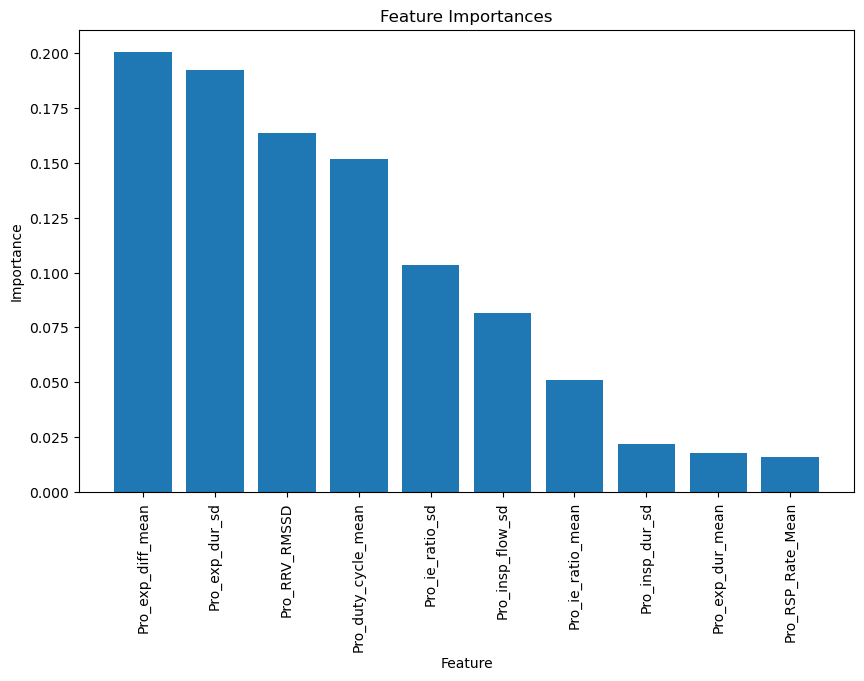


**Supplementary Figure 5**

*Graph of the importance of each feature for the Accelerometer model*


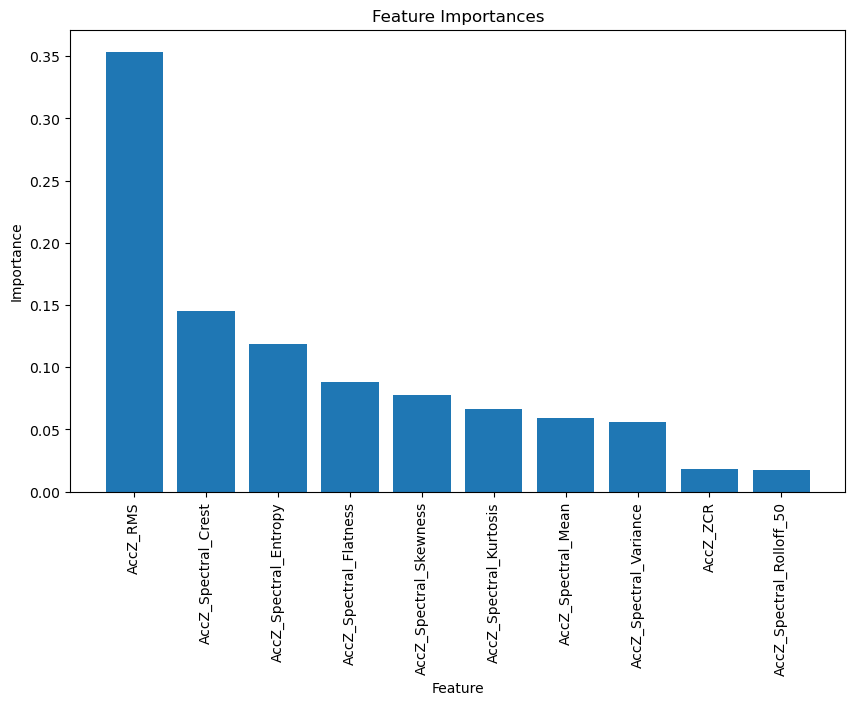


*Note*. For the device used, Z-axis is equivalent to the sagittal axis (perpendicular to the upper-sternum surface).

# Description of the structure of the GitHub repository with scripts and example datasets

The main folders available in the repository (<https://github.com/melisasaygin/SpeechDetection>) include the following: *Machine Learning Pipeline*, *Feature Extraction*, *Speech Detection Models*, and *Between Methods Comparison using Test Set*. Except for one in .R, all scripts in the repository are in .ipynb format used for Jupyter Notebooks, an interface for the Python programming language.

Under the *Feature Extraction* main folder, there are three folders entitled *Accelerometer, Impedance*, and *RIP*. Within each, there is a script illustrating the steps to extract the features for the method. For example, within *Accelerometer* there is the script (*AccFeatureExtraction*.ipynb) showing how, starting from a raw accelerometer signal, the spectral and time-domain features were extracted. The script under *Impedance* was used to extract the features from the Z0 signal of both devices used in the study. The feature extraction scripts all start with some preprocessing of the raw data, which includes filtering, demeaning, and Fast Fourier transformation of signal segments for the Accelerometer method, and filtering followed by peak-trough detection for the respiratory (RIP and Impedance) methods. The uploaded scripts are already ran using an example dataset, and the outputs can be reviewed in the GitHub preview. The example data can be accessed via the zipped *Raw Data* folder. Note that this constitutes of the raw data of a participant who gave consent for their data to be uploaded to an open-access platform.

Under the main folder *Machine Learning Pipeline*, there is a folder per method (*2bandsRIP, Acc, Impedance(5fs), Impedance(Core), ThoraxRIP*). Within a method’s folder, its mutual information gain based feature selection script can be found as the file ending with *Feature Selection*.ipynb. It goes over how the ten features for a given method were selected. Each method’s folder also has four other scripts whose names start with *Nested Cross-Validation*, containing the code for running the nested cross-validations using the ten features previously selected. One nested cross-validation script exists per each of the four learning algorithms (Gradient Boosting, XGBoost, Random Forest, Logistic Regression). One of these four scripts (i.e., one with the best-performing learning algorithm for a given method) also contain the training of the best final model of that method as a subsequent step. This document’s name starts with *Nested Cross-Validation + Best Model*, and it is possible to see the particular hyperparameters (as in Section 3) entered while training the final model, as well as the performance indices of this model as tested on the out-of-sample test set.

Under the main folder *Between Methods Comparison using Test Set*, the R script used to carry out the DeLong’s test between the performances (AUCs) of the different methods’ models is present. Under the main folder *Speech Detection Models*, intended for the deployment and straightforward use of the final models, there are four folders: *Accelerometer, Impedance, Thorax RIP,* and *Others.* Within a folder, there is the saved model in pickle format (e.g., *acc_model.pkl*) along with an associated simplified script (e.g., *AccModel.ipynb*) that calls the model to output predictions. We also provide a sample dataset that can be used to gain a practical understanding of how the prediction pipeline works. The steps on how to use the Thorax RIP, Impedance, and Accelerometer models are detailed in the next section. Within each script of the repository, there is commentary to guide the user. Improvements, additions, and other modifications to the repository will be documented in the README.md on the main page.

# Instructions to use the methods for speech detection

Below, we go over the steps to use the Thorax Belt Respiratory Inductance Plethysmography (Thorax RIP), Thoracic Impedance (ImP), and Sternal Accelerometer (Acc) models on new data. The files mentioned can be accessed via the GitHub repository. We strongly recommend to have the repository open and follow the directions as you read through the steps. Prior to trying out with own data, go through the steps using the data as available on the repository. If you will need to run the script yourself, you can download Anaconda Navigator to access the Jupyter Notebook environment.

## 6.1 Speech detection using respiratory inductance plethysmography

To implement the thorax RIP method’s trained machine learning model, one should first verify that increases in amplitude of raw thorax respiration correspond to inspiration, and if not the case, invert the signal around the x-axis. Add a new column, *Task_Labels_2*, to the raw data to label the 30-sec segments for which you want to do speech detection with distinct labels (e.g., naming all the rows representing a 30-sec of interest with 1, naming the rows of the next segment of interest with 2). Then, the signal needs to be partitioned into the segments of interest (i.e., into smaller data frames) *together* with 5 seconds before and after each 30-sec segment acting as buffer periods. For elimination of high-frequency interference and baseline drift, signal filtering of each 40-sec segment should be followed by the peak and trough detection of the breathing waveforms. We suggest the use of *scipy* or another method under the package *neurokit2,* though other packages or software may be used*.* The parameters of the filter and the peak-trough detection function *should* be adjusted (i.e., between-persons, within-person according to varying heart rate or activity intensity) to optimize accuracy. An error of 5% (i.e., incorrectly labeled peaks and troughs) is acceptable.

Then, using the respiratory amplitude values and the peak-trough indices, the ten features needed by the Thorax RIP model (listed in Supplementary Table 10) should be extracted for every 30-sec segment of interest, excluding the buffer periods. The script used for extracting the features can be found by clicking on the main folder *Feature Extraction*, then to *RIP*, as *RIPThoraxFeatureExtraction*.ipynb. The script starts with an example raw respiration signal, filters and performs peak-trough detection over 40-sec (30-sec plus buffer periods), only then calculates the features for every segment of interest and concatenates the features per segment. You can bypass these steps if the cleaned signal with peaks and troughs are obtained. In that case, the data should be brought to the format (explained below) needed by the feature extraction portion.

As can also be seen in *RIPThoraxFeatureExtraction*.ipynb, the data frame of each segment (with the buffer periods) is placed into a list object. Each individual data frame within the list has a column named as *RSP_Clean* including the filtered signal values, a column *RSP_Peaks* that has a 1 whenever there is a peak (otherwise 0), a column *RSP_Troughs* that has a 1 whenever there is a trough (otherwise 0), and *Task_Label_2*. Each data frame is named as the label of that segment (e.g., if a data frame is named as 1, most of the *Task_Label_2* will have 1s and the buffer periods before and after with no or other label). After having this list of data frames, enter your task labels (e.g., 1, 2, 3) in the cell provided, and run it: features will be extracted per segment, and correctly named with its variable name (e.g., mean duty cycle column named as *mean_duty_cycle*). The data frame with each row representing a 30-sec segment of interest (i.e., for which you would like to perform speech activity detection), and each column representing a feature can be saved as an .xlsx file.

Following, go to the main folder *Speech Detection Models*, then to *Thorax RIP*. There, download both *thorax_model.pkl* and *ThoraxRIPModel.ipynb*, and place these in the same folder (e.g., on your Desktop) with your .xlsx file. Open *ThoraxRIPModel*.ipynb, enter your .xlsx file’s name in the designated place of the first cell, and run each cell. The final output will be a list of 1s and 0s indicating speech presence or lack thereof per segment of interest, in chronological order.

## 6.2 Speech detection using impedance pneumography

To implement the impedance model, the same series of steps are followed as with the abovementioned steps for thorax-RIP, with several differences. Again, the *Task_Label_2* column should be added to the raw signal column to match signal portions with the segments of interest. For instance, if the sampling rate of the Z0 impedance signal being used is 250 Hz, each segment of interest will have 7500 rows of raw data. The buffer period for impedance is 7 seconds before and after each 30-sec segment. For every 44-sec segment, new data frames should be created, and filtering and peak-trough detection should be done. To find the feature extraction script for the method, go to the folder *Feature Extraction*, and click *Impedance* to access *ImpedanceFeatureExtraction*.ipynb. After extracting the ten features (listed in Supplementary Table 12) per each 30-sec of interest, save as an .xlsx file.

Then, go to the main folder *Speech Detection Models*, and then to *Impedance*. Download both *impedance_model.pkl* and *ImpedanceModel.ipynb*, and place these in the same folder with your .xlsx file containing the features. Open *ImpedanceModel*.ipynb, enter your file’s name in the first cell, and run each cell. As the last output, you will receive a list of 0s and 1s indicative of speech activity.

## 6.3 Speech detection using accelerometers on sternum

Collect the raw accelerometer data in the sagittal axis using a VU-AMS Core or another upper sternum positioned accelerometer unit that features the same accelerometer as the one used in the mentioned device. Refer to Discussion section for the specifications. Add a new column named *Task_Label_2* to the dataset containing the raw accelerometer signal along the sagittal axis. This column should specify the segments intended for speech detection. As the sampling rate for the accelerometer is 1000 Hz, there would be 30000 rows for each of the segments you label (e.g., 30000 rows of 1, 30000 rows of 2). Neither separate into smaller data frames nor add buffer periods (as was done in the previous methods). Enter this data frame of two columns to the feature extraction script of the Acc method. This can be found under the folder *Feature Extraction*, and then clicking to *Accelerometer*, as *AccFeatureExtraction*.ipynb. Write out the label of the segments (e.g., 1, 2, 3) into the list of tasks in the designated section. Run the cells, and save the .xlsx file containing the features derived. Then, go to the main folder *Speech Detection Models*, then to *Accelerometer*. Download both *acc_model.pkl* and *AccModel.ipynb*, and place these in the same folder with your .xlsx file containing the features. Open *AccModel*.ipynb, enter your file’s name in the first cell, and run each cell. As the output of the last cell, you will receive a list of 0s and 1s.

# Potential use of the methods for speech detection

## 7.1 Speech detection using respiratory inductance plethysmography

RIP typically has both a thoracic and abdominal belt, is accepted as the gold standard for ambulatory respiration monitoring, and a number of procedures and analytical techniques have been developed for calibrating RIP amplitude readings to absolute volume (milliliters) using either a spirometer, pneumotachometer, or a fixed-volume spirobag (Darling-White, 2022; Grossman et al., 2010). Thus, the RIP model can be especially suitable for ambulatory studies interested in continuously capturing the tidal volume, as well as those examining other respiratory changes in response to psychosocial phenomena. A specific example would be a study measuring cardiac vagal activity by correcting peak-valley respiratory sinus arrhythmia (RSA) values for tidal volume (mL) and respiratory rate (Ritz & Dahme, 2006), in response to momentary perceived affect. Tidal volume estimations are improved when using both the thorax and abdomen belts (Darling-White, 2022; Konno & Mead, 1967). However, using both belts did not increase any of AUC, accuracy, sensitivity, or specificity as compared to a thorax only RIP model for speech classification. Here, using only the thorax band will not just reduce the data processing effort but will also enhance the accuracy of speech detection.

In the ambulatory monitoring market, wearables measuring the mechanical expansion of the thorax through a strain gauge sensor (e.g., Zephyr BioHarness 3 by Zephyr Technology Corporation, eq02+ LifeMonitor by Equivital), piezoresistive, or piezoelectrical sensors are relatively more common than using RIP. The Thorax-RIP model is expected to perform well on data collected with these devices, though the prediction accuracy may be reduced. Steps on how to implement the Thorax RIP model to perform speech detection are outlined in the Supplementary, section 6.1.

## 7.2 Speech detection using impedance pneumography

The thoracic impedance based detection model is suitable for research on stress reactivity interested in capturing autonomic nervous system activity and using impedance cardiography (e.g., VU-AMS 5fs, VU-AMS Core, MindWare Mobile) to do so. When used in conjunction, impedance cardiography (ICG) and electrocardiography not only provide a widely used index for cardiac sympathetic control (other branches of the sympathetic efferent outflow around the body substantially differ in their level of activity; for a review see Morrison, 2001), the preejection period, but also a proxy respiration signal (Z0) that can be used to index peak-valley RSA (De Geus & Gevonden, 2022), an index that has been used to indicate cardiac parasympathetic control. By already having the Z0 signal from ICG, such psychophysiology studies can detect speech as a confounding factor at no additional burden.

The method was validated for two different devices, and all indices of AUC, accuracy, sensitivity and specificity for both were always greater than 80% except for the sensitivity of the VU-AMS Core device in the nested cross-validation, which was very close with 79.3%. The features for the models overlap, and the two most important features, mean first difference of exhalation and standard deviation of expiratory duration, were the same. The reason VU-AMS Core might have performed less well than other methods in this comparison may have been signal degradation due to a high-frequency interference in its Z0 signal when used simultaneously with VU-AMS 5fs. Impedance cardiography devices each have their own current source and during simultaneous use these may interfere with each other, which is a problem specific to head-to-head comparisons such as in this experiment. Recordings taken with the VU-AMS Core did not show any high-frequency interference in its Z0 signal when it was the only ICG device. Since the interference only occurred one way, we suggest the use of the best model from the 5fs method to detect speech through thorax impedance data from any ambulatory ICG device, including the VU-AMS Core.

## 7.3 Speech detection using accelerometers on sternum

Use of accelerometry (Acc) provided higher accuracy, AUC, sensitivity, and specificity than the respiration-based methods and required significantly less processing effort as an intermediary peak-trough identification step is not needed. This method is ideal for studies aimed at detecting speech continuously (i.e., not for predetermined windows) to label periods of acute stress, higher listening effort, or assess ın-person social interaction levels. If using VU-AMS Core, which records both the sternal Acc and thoracic impedance, the Acc model should be preferred for speech detection. The VU-AMS Core combines the sternal triaxial accelerometry unit with ECG and ICG recording because its intended use is primarily to record autonomic nervous system activity. If speech is the primary focus, just using a sternal accelerometer without any electrodes will also be sufficient and less burdensome to participants.

In order to use the Acc method, the triaxial accelerometry unit needs to be placed just inferior of the suprasternal notch. The brand and type of the accelerometry unit validated in the current paper as part of the VU-AMS Core device was InvenSense 16G accelerometry unit, each axis sampling at 1000 Hz. The z-direction of the accelerometer we used (AccZ) refers to the sagittal (anterior-posterior) plane of the body. The model only needs features derived using the Acc signal in the sagittal axis.

# Rationale for bypassing the feature normalization step

Prior to training a machine learning model, it is common practice to normalize the extracted features. The reasoning behind this is that if features are not standardized, those with larger scales can disproportionately influence the machine learning algorithm, which can result in a considerable decrease in the model’s prediction accuracy when tested on new data. However, we did not implement feature normalization in any part of our machine pipeline. The reasoning is twofold.

First, the lab data collected consists of 33.3% speech and 66.6% silent segments per participant, and if we were to normalize the features within-person across these segments, the trained models could develop an inherent bias. The proportion of speech to silence periods in daily life will inevitably be different from this 1:2 ratio and will vary from individual to individual and day to day. It would also be unclear how a researcher should standardize the data per participant to be able to use the speech detection models effectively. Thus, normalizing the features could virtually eliminate the chances of the trained machine learning models generalizing to real-world data.

Second, the particular machine learning algorithms we used – Gradient Boosting, XGBoost, and Random Forest— are inherently robust to features having different scales. In contrast, algorithms such as Support Vector Machines and k-Nearest neighbors strongly rely on feature normalization for optimal model performance. Thus, in the case of the present study, it does not constitute a problem to skip this normalization step. Instead, it maximizes the chances that the deployed models show better validity and practicality when applied to daily life data.

# Individual differences in model performance: AUC, Accuracy, Sensitivity, Specificity

We investigated whether model performance holds at the individual level and whether it changes based on the demographics of age, sex, and body mass index. As we have implemented a leave-one-out approach in the nested cross-validations, each outer loop’s performance metrics represents those obtained when tested on a new held-out participant. In Supplementary Figures 6-10, we provide the distribution of individual performance metrics across the outer loops of the (best) nested cross-validation per method. We then proceeded to correlate these performance indices with age, sex, and body mass index. We provide the correlation heatmaps in Supplementary Figures 11-15. As stated in the paper itself, only the correlation between biological sex and specificity for the impedance method was significant. Finally, we look into how well the best single model per method performs for the subgroups of low BMI, high BMI, male, and female both in the entire sample and only in the test set (see below the Supplementary Tables 15-18).

**Supplementary Figure 6**

*Histogram of individual performance metrics for the nested cross validation of Thorax-RIP method*


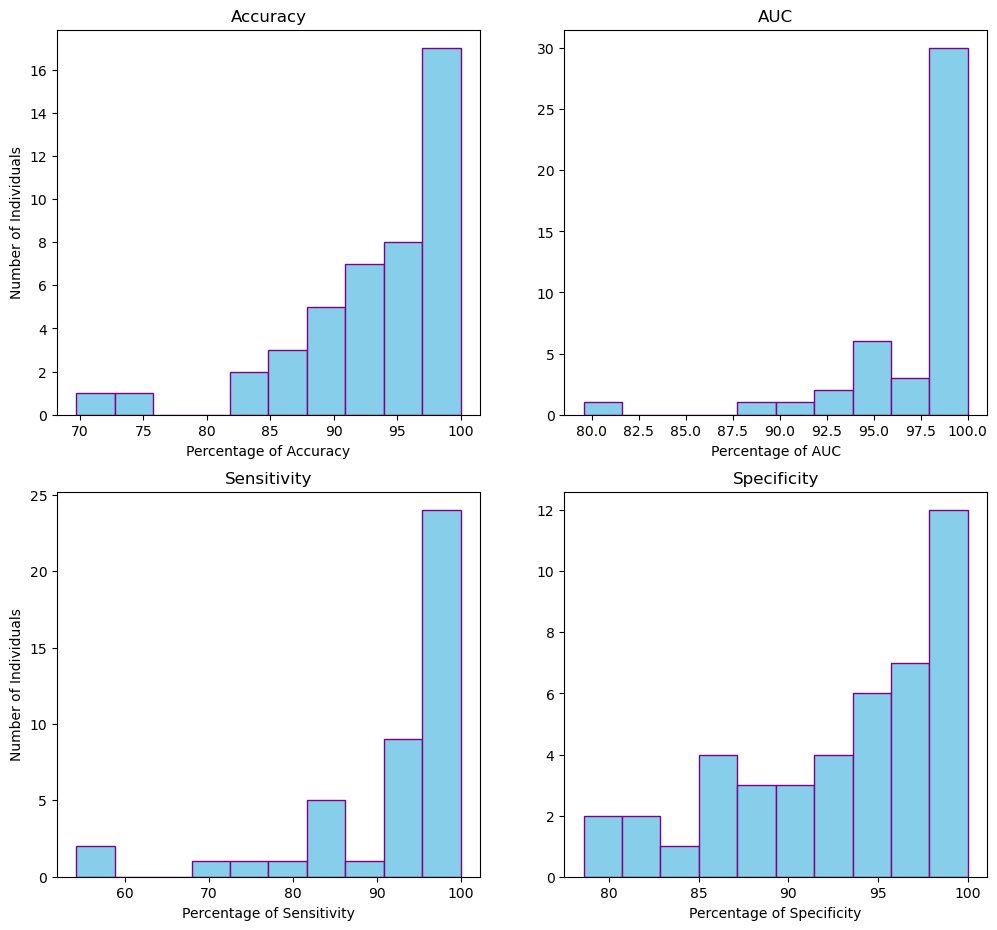


**Supplementary Figure 7**

*Histogram of individual performance metrics for the nested cross validation of 2-bands RIP method*


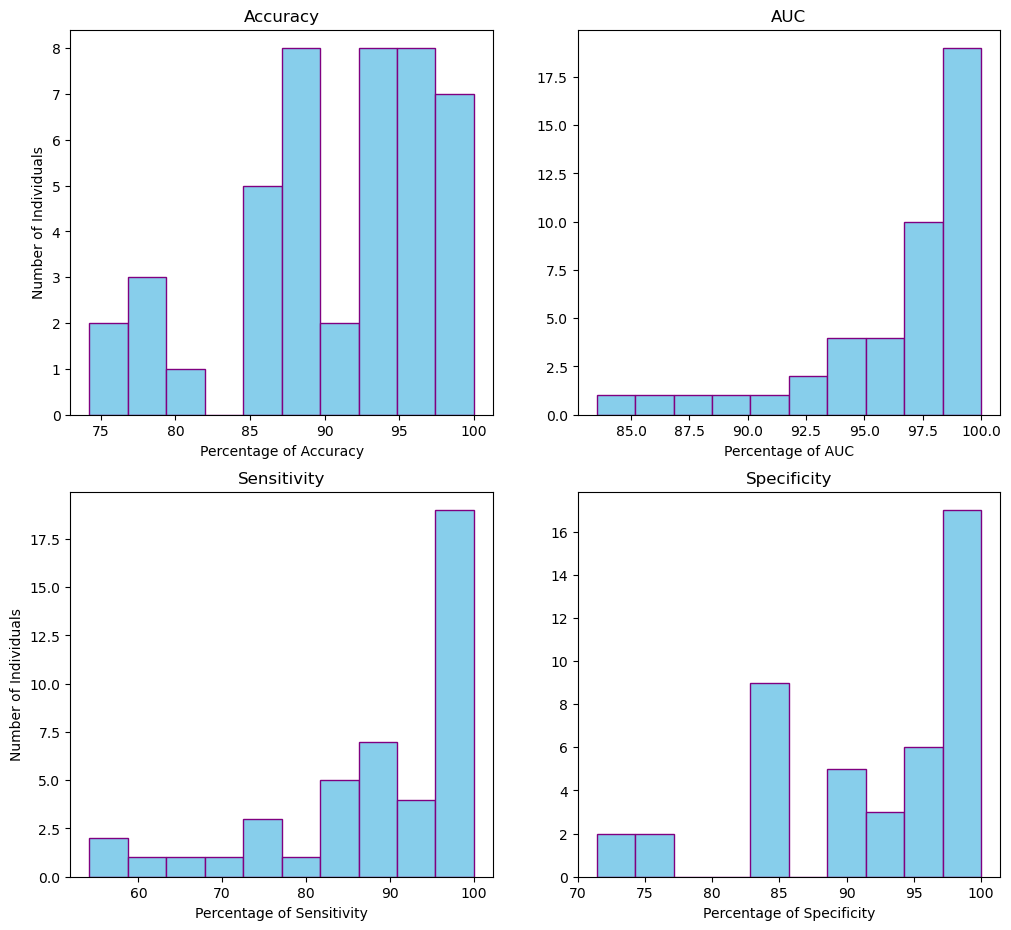


**Supplementary Figure 8**

*Histogram of individual performance metrics for the nested cross validation of Impedance (VU-AMS 5fs) method*

**
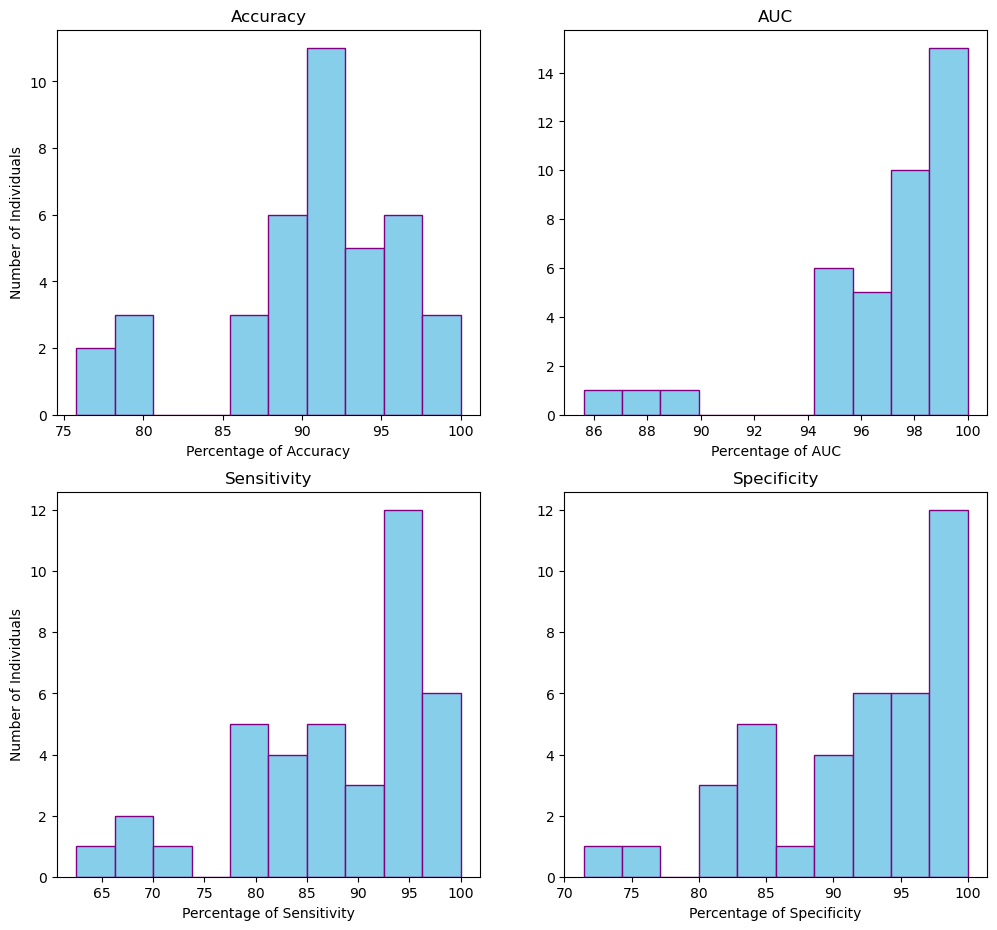
**

**Supplementary Figure 9**

*Histogram of individual performance metrics for the nested cross validation of Impedance (VU-AMS Core) method*

**
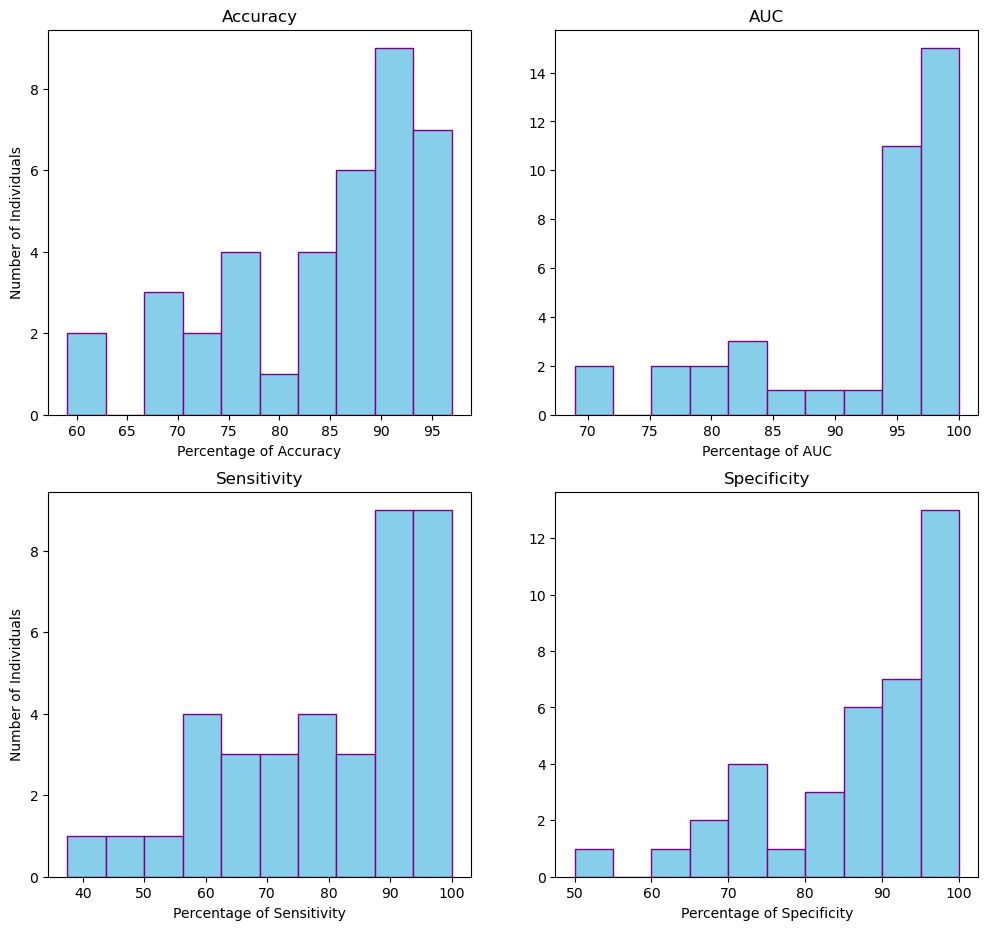
**

**Supplementary Figure 10**

*Histogram of individual performance metrics for the nested cross validation of Accelerometer method*

**
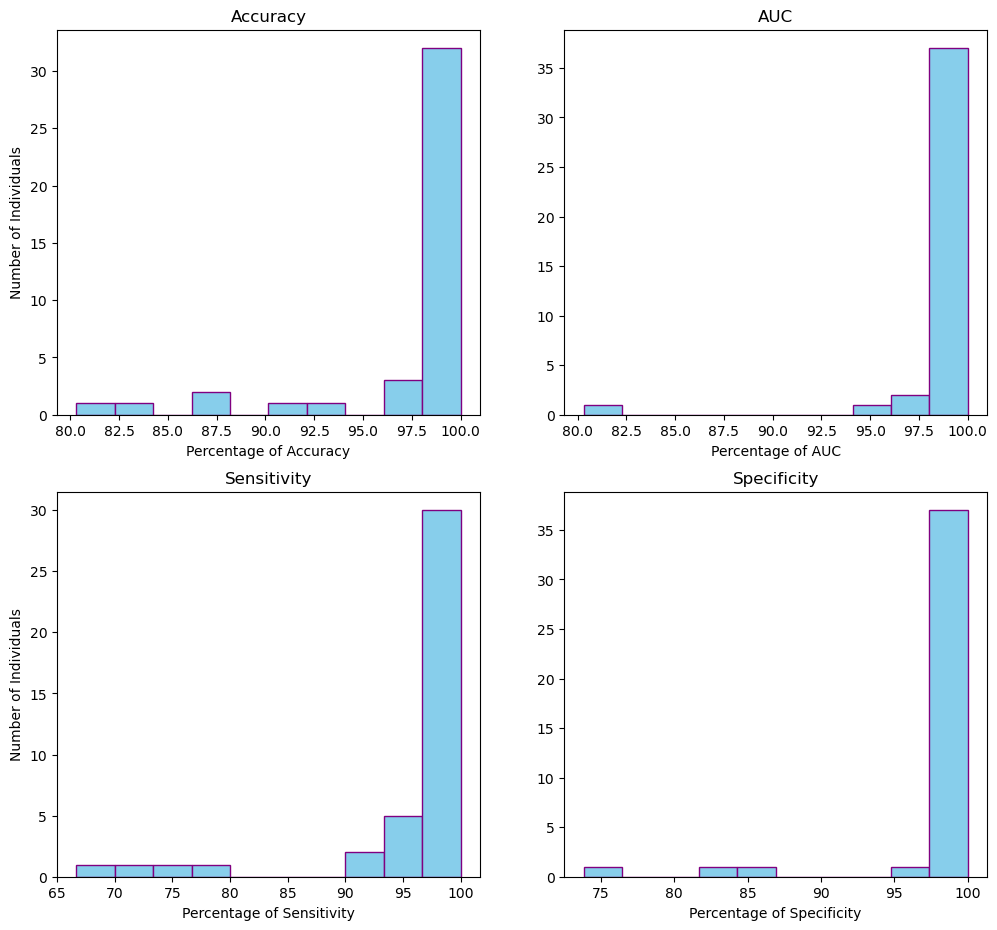
**

**Supplementary Figure 11**

*Correlation matrix across nested CV outer loop performance indices, sex, age, BMI:* Thorax RIP


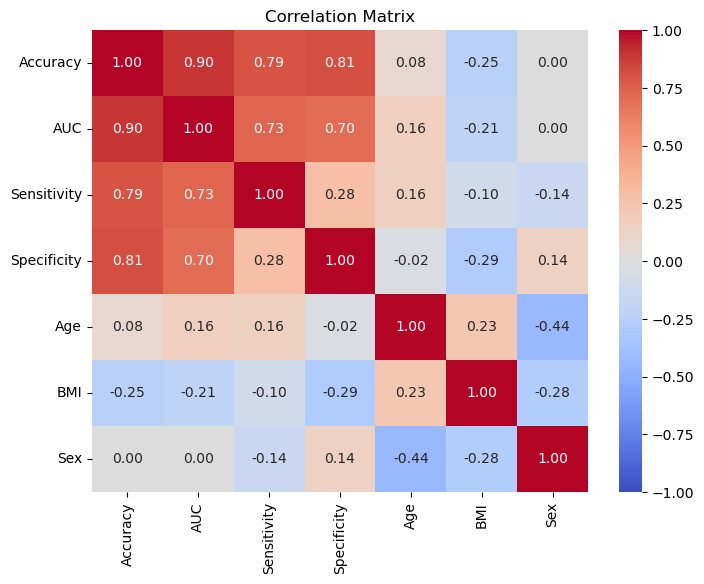


**Supplementary Figure 12**

*Correlation matrix across nested CV outer loop performance indices, sex, age, BMI: 2-bands (thoracoabdominal) RIP*


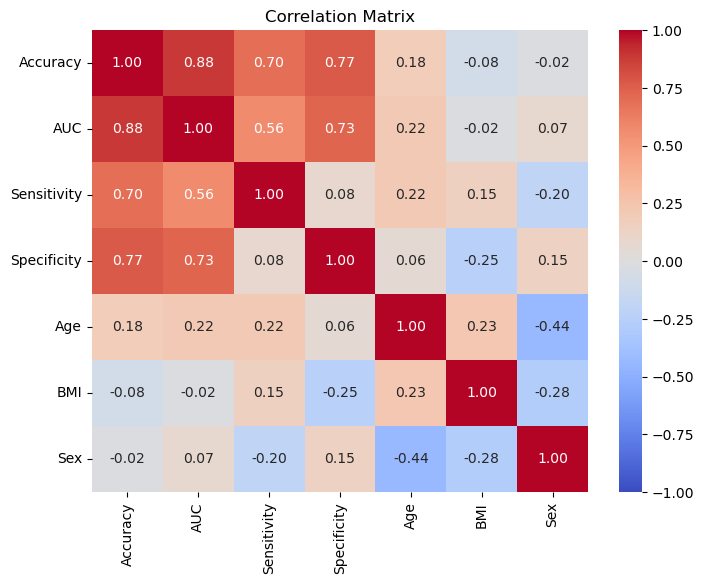


**Supplementary Figure 13**

*Correlation matrix across nested CV outer loop performance indices, sex, age, BMI: Impedance (VU-AMS 5fs)*


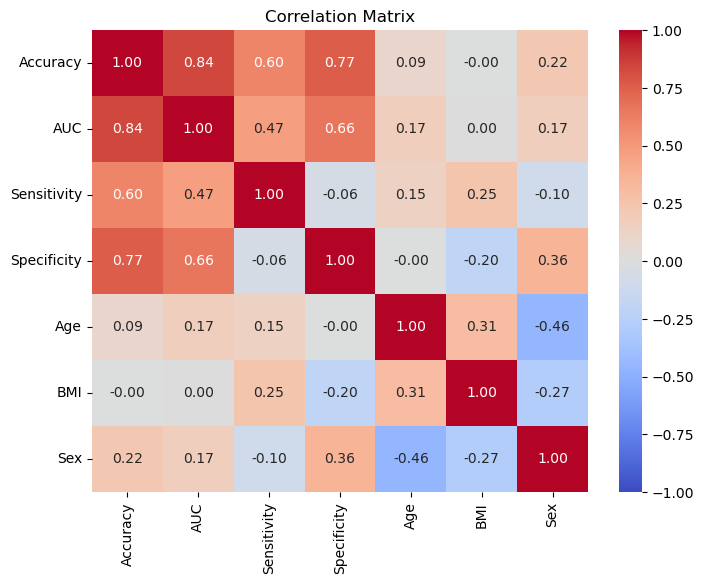


**Supplementary Figure 14**

*Correlation matrix across nested CV outer loop performance indices, sex, age, BMI: Impedance (VU-AMS Core)*


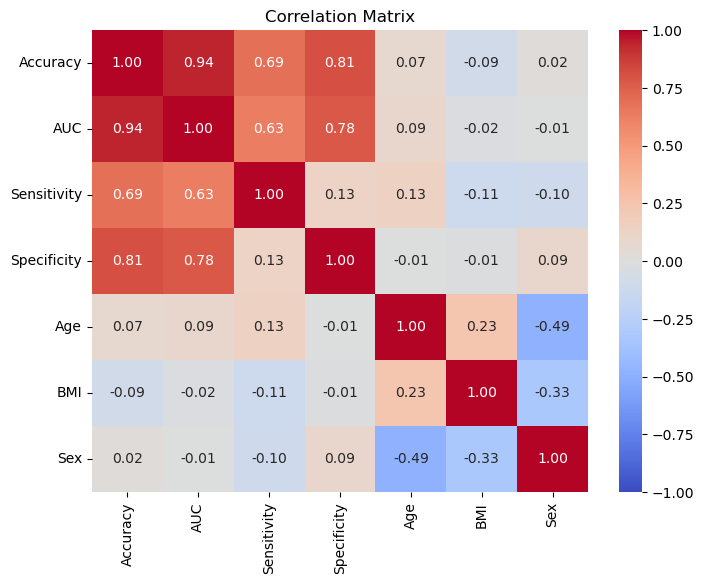


**Supplementary Figure 15**

*Correlation matrix across nested CV outer loop performance indices, sex, age, BMI: Accelerometer*

**
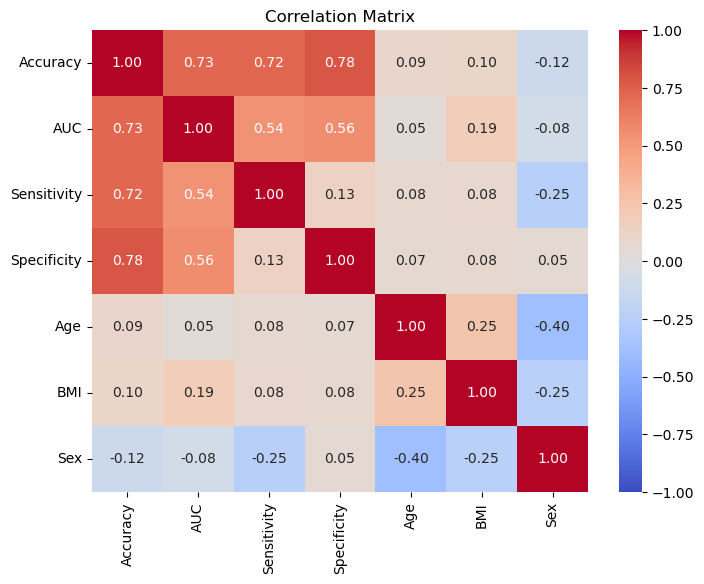
**

**Supplementary Table 15**

*AUC metric of subgroups for sex and body mass index*

|  | Female | Male | Low BMI | High BMI |
| --- | --- | --- | --- | --- |
|  | Full Sample |  |  |  |
| Thorax RIP | 99.6% | 99.8% | 99.7% | 99.5% |
| 2-bands RIP | 99.5% | 99.9% | 99.7% | 99.6% |
| Impedance (VU-AMS 5fs) | 99.6% | 99.7% | 99.7% | 99.5% |
| Impedance (VU-AMS Core) | 96.6% | 96.9% | 96.6% | 96.8% |
| Acc | 99.9% | 100% | 100% | 99.9% |
|  | Test Set Only |  |  |  |
| Thorax RIP | 99.0% | 99.6% | 98.8% | 99.2% |
| 2-bands RIP | 98.4% | 99.6% | 98.0% | 98.8% |
| Impedance (VU-AMS 5fs) | 97.7% | 98.2% | 97.9% | 97.9% |
| Impedance (VU-AMS Core) | 95.8% | 94.3% | 96.1% | 95.6% |
| Acc | 99.7% | 99.9% | 99.8% | 99.4% |

**Supplementary Table 16**

*Accuracy metric of subgroups for sex and body mass index*

|  | Female | Male | Low BMI | High BMI |
| --- | --- | --- | --- | --- |
|  | Full Sample |  |  |  |
| Thorax RIP | 97.1% | 97.9% | 97.3% | 97.2% |
| 2-bands RIP | 97.3% | 98.4% | 97.6% | 97.4% |
| Impedance (VU-AMS 5fs) | 97.8% | 99.2% | 98.3% | 97.8% |
| Impedance (VU-AMS Core) | 89.4% | 90.6% | 89.3% | 90.0% |
| Acc | 99.7% | 99.7% | 99.7% | 99.7% |
|  | Test Set Only |  |  |  |
| Thorax RIP | 95.6% | 98.5% | 93.6% | 97.5% |
| 2-bands RIP | 94.4% | 98.5% | 93.2% | 96.0% |
| Impedance (VU-AMS 5fs) | 91.8% | 93.9% | 91.3% | 92.4% |
| Impedance (VU-AMS Core) | 87.5% | 84.8% | 87.5% | 87.1% |
| Acc | 98.8% | 95.5% | 98.1% | 98.7% |

**Supplementary Table 17**

*Sensitivity metric of subgroups for sex and body mass index*

|  | Female | Male | Low BMI | High BMI |
| --- | --- | --- | --- | --- |
|  | Full Sample |  |  |  |
| Thorax RIP | 96.1% | 98.3% | 95.8% | 97.3% |
| 2-bands RIP | 96.2% | 99.0% | 96.2% | 97.5% |
| Impedance (VU-AMS 5fs) | 97.1% | 98.6% | 96.7% | 98.0% |
| Impedance (VU-AMS Core) | 85.5% | 90.5% | 84.4% | 88.7% |
| Acc | 99.7% | 100% | 99.7% | 99.8% |
|  | Test Set Only |  |  |  |
| Thorax RIP | 94.4% | 100% | 90.6% | 97.9% |
| 2-bands RIP | 92.6% | 100% | 89.6% | 95.8% |
| Impedance (VU-AMS 5fs) | 89.4% | 91.7% | 84.4% | 93.1% |
| Impedance (VU-AMS Core) | 82.4% | 79.2% | 77.1% | 85.4% |
| Acc | 98.6% | 100% | 97.9% | 99.3% |

**Supplementary Table 18**

*Specificity metric of subgroups for sex and body mass index*

|  | Female | Male | Low BMI | High BMI |
| --- | --- | --- | --- | --- |
|  | Full Sample |  |  |  |
| Thorax RIP | 97.7% | 97.6% | 98.2% | 97.2% |
| 2-bands RIP | 97.9% | 98.0% | 98.4% | 97.4% |
| Impedance (VU-AMS 5fs) | 98.2% | 99.5% | 99.2% | 97.6% |
| Impedance (VU-AMS Core) | 91.6% | 90.6% | 92.0% | 90.8% |
| Acc | 99.8% | 99.5% | 99.7% | 99.6% |
|  | Test Set Only |  |  |  |
| Thorax RIP | 96.3% | 97.6% | 95.2% | 97.2% |
| 2-bands RIP | 95.5% | 97.6% | 95.2% | 96.0% |
| Impedance (VU-AMS 5fs) | 93.1% | 95.2% | 95.2% | 92.1% |
| Impedance (VU-AMS Core) | 90.5% | 88.1% | 93.5% | 88.1% |
| Acc | 98.9% | 92.9% | 98.2% | 98.4% |

References

Darling-White, M. (2022). Comparison of Respiratory Calibration Methods for the Estimation of Lung Volume in Children With and Without Neuromotor Disorders. *Journal of Speech, Language, and Hearing Research*, *65*(2), 525–537. https://doi.org/10.1044/2021_JSLHR-21-00333

De Geus, E. J. C., & Gevonden, M. J. (2022). Acquisition and Analysis of Ambulatory Autonomic Nervous System Data. In *Mobile Sensing in Psychology: Methods and Applications*. Guilford Publications Inc.

Grossman, P., Wilhelm, F. H., & Brutsche, M. (2010). Accuracy of ventilatory measurement employing ambulatory inductive plethysmography during tasks of everyday life. *Biological Psychology*, *84*(1), 121–128. https://doi.org/10.1016/j.biopsycho.2010.02.008

Konno, K., & Mead, J. (1967). Measurement of the separate volume changes of rib cage and abdomen during breathing. *Journal of Applied Physiology*, *22*(3), 407–422. https://doi.org/10.1152/jappl.1967.22.3.407

Morrison, S. F. (2001). Differential control of sympathetic outflow. *American Journal of Physiology-Regulatory, Integrative and Comparative Physiology*, *281*(3), R683–R698. https://doi.org/10.1152/ajpregu.2001.281.3.R683

Ritz, T., & Dahme, B. (2006). Implementation and Interpretation of Respiratory Sinus Arrhythmia Measures in Psychosomatic Medicine: Practice Against Better Evidence?: *Psychosomatic Medicine*, *68*(4), 617–627. https://doi.org/10.1097/01.psy.0000228010.96408.ed
